# Evolutionary Refinement of Mitochondrial and Plastid Targeting Sequences Coincides with the Late Diversification of Land Plants

**DOI:** 10.1093/molbev/msaf240

**Published:** 2025-09-23

**Authors:** Parth K Raval, Carolina García García, Maria-Darline Somoano Sanchez, Sven B Gould

**Affiliations:** Institute for Molecular Evolution, Heinrich-Heine-University Düsseldorf, Düsseldorf 40225, Germany; Institute for Molecular Evolution, Heinrich-Heine-University Düsseldorf, Düsseldorf 40225, Germany; Institute for Molecular Evolution, Heinrich-Heine-University Düsseldorf, Düsseldorf 40225, Germany; Institute for Molecular Evolution, Heinrich-Heine-University Düsseldorf, Düsseldorf 40225, Germany

**Keywords:** protein sorting, organelles, terrestrialization, plastid, mitochondria

## Abstract

Plastids and mitochondria are key to plant survival and adaptation. The evolutionary progress of land plants (embryophytes) witnessed gene and genome duplications, and the expansion of organelle-localized proteins. To deal with the increase of nuclear-encoded proteins, targeting to and import by the mitochondrion and plastid are known to have adapted in multiple ways. It included the addition of entirely new import channels and lineage-specific import receptors. Through comparative genomics and experimental biology, we uncover further changes in the organelle import machineries. Their evolution likely served to enhance the rate of protein import and improve its physiological regulation, e.g. via interactions between the import channel and respiratory complex. On the cargo side, nuclear-encoded N-terminal targeting sequences of mitochondrial targeting peptide (TP) and plastidal (pTPs) proteins have diverged in their charge via a preference for phosphorylatable amino acids (AA) (adding negative charges after phosphorylation) and an avoidance of positive charges in the pTPs, which is most evident in eudicots. Using *Chlamydomonas* and *Marchantia*, we experimentally underscore that the evolved TP divergence prevents mis-sorting between mitochondria and plastids. In accordance with the increase in phosphorylatable AA in the pTPs, we pinpoint the embryophytic origin of a membrane-anchored phosphatase, PAP2, which is associated with targeting sequence processing. On the whole, we propose a revised model for the evolution of plant organelle protein import from algae to angiosperms, which facilitated the flourishing of this lineage on land.

## Introduction

Mitochondria and plastids, two key compartments of photosynthetic eukaryotes, originate from Gram-negative bacteria (Keeling 2010 ; Archibald 2015; Martin et al. 2015), and part of their transformation from a free-living organism to an organelle includes the transfer of genetic information to the host nucleus (Timmis et al. 2004). A plethora of core functions they perform (Hatefi 1985; Eberhard et al. 2008; Wang and Youle 2009; Mesmin 2016; Hölzl and Dörmann 2019) hence hinge upon cytosolic protein biosynthesis and the subsequent import of proteins across a pair of membranes (Scotti et al. 2000; Hewitt et al. 2011; Day et al. 2014; Diederichs et al. 2020; Richardson and Schnell 2020). Protein import complexes, consisting of dozens of subunits, serve as a necessary gateway to all organelle functions and, by that, they affect fitness and macroevolutionary trends (Leebens-Mack et al. 2019; Knopp et al. 2020). Protein translocation across organelle membranes comprises an interplay of targeting sequences, chaperones, transport channels, and also kinases and phosphatases (Richardson and Schnell 2020; Schulte et al. 2023; den Brave et al. 2024). Owing to the challenge of simultaneous protein sorting between two organelles of endosymbiotic origin—which is based on very similar principles (Garg and Gould 2016; McKinnon and Theg 2019; Knopp et al. 2020)—this process is necessarily more complex in algae and plants and has likely been under a pertinent selection pressure since plastid origin.

The emergence of major archaeplastidal lineages such as the Chloroplastida or Embryophyta witnessed the expansion of organelle proteomes, which likely improved the ability to respond to abiotic stresses (Heinnickel and Grossman 2013; de Vries and Archibald 2018; Knopp et al. 2020; Raval et al. 2024). Translocation across the correct organellar membranes is key for these proteins to be able to perform their function. The main import channel of the outer chloroplast membrane in Chloroplastida, TOC75, likely originated through the duplication of the original import channel OEP80, and allowed for a division of labor between the two (Knopp et al. 2020). Concomitant with the origin of TOC75 in the green lineage was the loss of an N-terminal phenylalanine—and, to a lesser degree, other bulky amino acids (AA) such as W, Y, or L—based targeting motif (Wunder et al. 2007; Köhler et al. 2015; Knopp et al. 2020) which acted as the first point of contact to discriminate between mitochondrial and plastid cargo in rhodophyte and glaucophyte algae, apart from the features downstream of this motif that further conferred organelle specificity (Steiner et al. 2005; Gould et al. 2006; Gruber et al. 2007). We argue that the loss of this first point-of-contact at least partly compromised the initial identification of cargo at the outer organelle membranes (e.g. for a subset of proteins that strongly depended on F-based differentiation between plastids and mitochondria) and paved the way for additional mechanisms for import specificity to evolve. The expansion of organelle proteomes in land plants (Raval et al. 2024) likely provided a further positive feedback loop for the selection of a more flexible, yet also accurate cargo recognition.

Several strategies ensure correct protein sorting in the land plant models studied, among which N-terminal targeting sequences are one. The literature refers to such targeting sequences with various denominations, including presequence, transit sequence, targeting signal, or targeting peptide (TP). Mitochondrial TPs (mTPs) of matrix proteins are on average 50 AA long, while those of plastid proteins (pTPs) are usually longer, with some reaching up to 100 AA (Kim and Hwang 2013). mTPs and pTPs can vary substantially across proteins and species, and although some signature secondary structures are attributed to mTPs and pTPs (Cramer et al. 1990; Wang and Weiner 1993), detailed differences in secondary structures are not that well defined, and neither are all their physio-chemical properties (Lee et al. 2019; Caspari et al. 2023). One critical physicochemical property that aids cells in distinguishing cargo concerns charge differences, which are apparent even when considering only the first 20 AA of TPs (Garg and Gould 2016). In *Arabidopsis*, the first 20 AA of mTPs (approximately one-third of the entire TP) are characterized by a higher positive charge, which is typical for nuclear-encoded mitochondrial matrix proteins in general, whereas those of the pTPs are enriched with serine residues (Bhushan et al. 2006; Lee et al. 2006a; Pujol et al. 2007; Ge et al. 2014). Phosphorylation of pTP residues, followed by dephosphorylation at the plastid outer membrane, regulates the import rate of some plastid cargo (May and Soll 2000) and simultaneously suppresses import by mitochondria of otherwise plastid-targeted proteins (Garg and Gould 2016). While in-silico modeling suggests that features such as the overall charge are sufficient to separate an mTP from a pTP (Ge et al. 2014; Garg and Gould 2016), more experimental support is needed (Bhushan et al. 2006; Holbrook et al. 2016; Lee et al. 2019). Considering the key role of the first 20 AA of TPs as the first point of contact, the causes and consequences of the substantial evolutionary shift from a highly conserved bulky AA-based motif to a serine-enriched motif are important to understand.

Current data regarding the interplay of targeting sequences and import components of mitochondria and plastids are largely restricted to the model system *Arabidopsis* and a few other angiosperms. A comprehensive picture of how conserved the interplay is across Archaeplastida or how changes might have influenced major transitions in algae and plant evolution is missing (de Vries et al. 2016). We do not yet fully understand how plastid proteins are exactly distinguished from mitochondrial ones, how dual-targeted cargo is selected, or what exact regulatory role the evident cytosolic phosphorylation and de-phosphorylation at the outer membranes of these organelles might play (Waegemann and Rgen Soll 1996; Martin et al. 2006; Garg and Gould 2016; Zhang et al. 2016; Law et al. 2018). Here, combining comparative genomics, phylogenetics and experimental biology in the chlorophyte *Chlamydomonas* and an early diverging land plant, the bryophyte *Marchantia*, we track the origins and evolution of the protein import components across the archaeplastidal lineage and characterize changes that improved accuracy, and likely the rate, of organelle protein import.

## Results

### Divergence of Mitochondrial and Plastid Targeting Sequences upon Terrestrialization

**Fig. 1.**
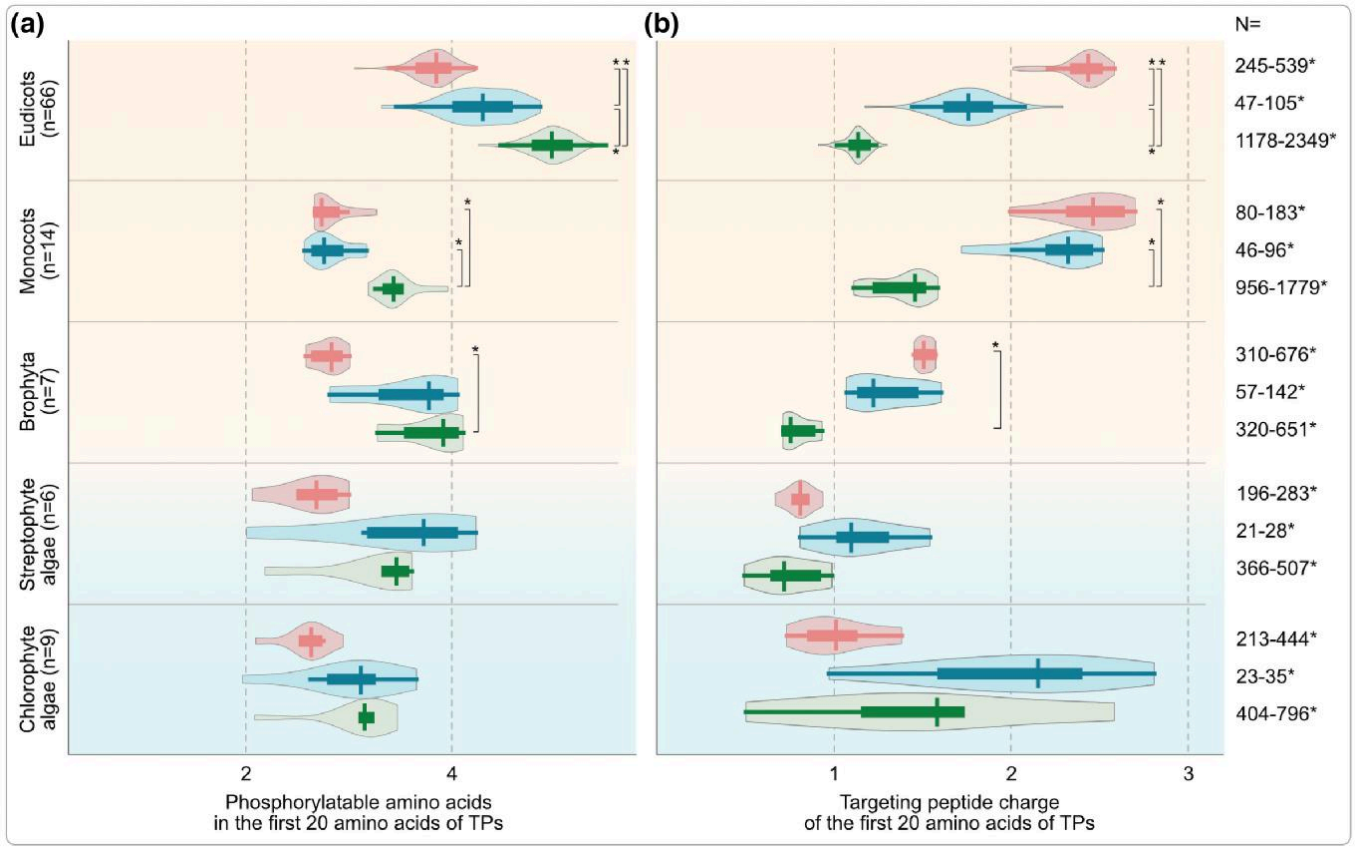
The evolution of AA charge across the TPs of photosynthetic eukaryotes. The total number of a) phosphorylatable AA (serine + threonine) and b) electric charge for each protein, in a given species, was calculated from the first 20 AA of N-terminal TPs of mitochondrial, plastid, and dual-targeted proteins. Mean of all proteins of a given organelle in a given species was treated as a representative and plotted for each clade within a violin plot. Each violin, therefore, represents organelle proteomes of an entire clade, with the top three representing land plants and the bottom two representing green algae. Within each violin plot, the vertical line represents the median, the thick horizontal line the interquartile range (from 25th to 75th percentile), and the trailing thin horizontal line represents the full data range. Multiple ANOVA (with Bonferroni correction for multiple comparisons) was performed across and within clades and organelles. Select statistically significant pairs are shown on the right side (asterisks), full statistics are summarized in the figure source data. The exact *N*, number of organelle TPs for a given species from which the mean was calculated to be included in the clade-violin plot, depended on a species; therefore, a range is shown for a given organelle and clade on the right side of each violin; n, number of species in a given clade violin are indicated on the left.

Membrane receptors and channels can recognize a majority of their cargo based on N-terminal TPs, the initial region (e.g. the first 20 AA) of which acts as the first point-of-contact at the organelle outer membrane, and it is also the region where major evolutionary changes occurred. Note that we only focus on the first 20 AA of the TPs for these reasons, but refer to them as “the features of TPs” for simplicity. To trace the evolutionary history of changes in physicochemical properties of the first 20 AA of TPs from algae to angiosperms, we analyzed the TPs of proteins from five major chloroplastidal lineages inferred (based on orthology with experimental proteomes) to be either mitochondria-, plastid-, or dual-targeted (mTP, pTP, or dTP, respectively; Table S1b, Fig. S1). For these inferred TPs, we plot average numbers of serine and threonine (phosphorylatable AA) and their electric charge for each of the five clades. In algae, the distributions of phosphorylatable AA and charge for mTP and pTP overlapped (no statistically significant differences), making mTP and pTP rather indistinguishable for these features at the beginning of the TPs (Fig. 1a and b). Land plants (monocots and eudicots in particular) differ and show similar values across species with a clear separation between mTPs and pTPs on two counts: the beginning of the pTPs is enriched in phosphorylatable AA (Ser and Thr) from streptophyte algae to eudicots (Fig. 1a) and the beginning of the mTPs is enriched with respect to a positive charge across the sequences analyzed (Fig. 1b). Angiosperms, both mono- and eudicots, showed the clearest separation. The values of these features in the TPs of eudicotic proteins imported by plastids and mitochondria (the dual targeted proteins) range between those with only a single destination, underscoring a compromise with respect to dually targeted proteins exemplified in eudicots (Fig. 1a and b).

**Fig. 2.**
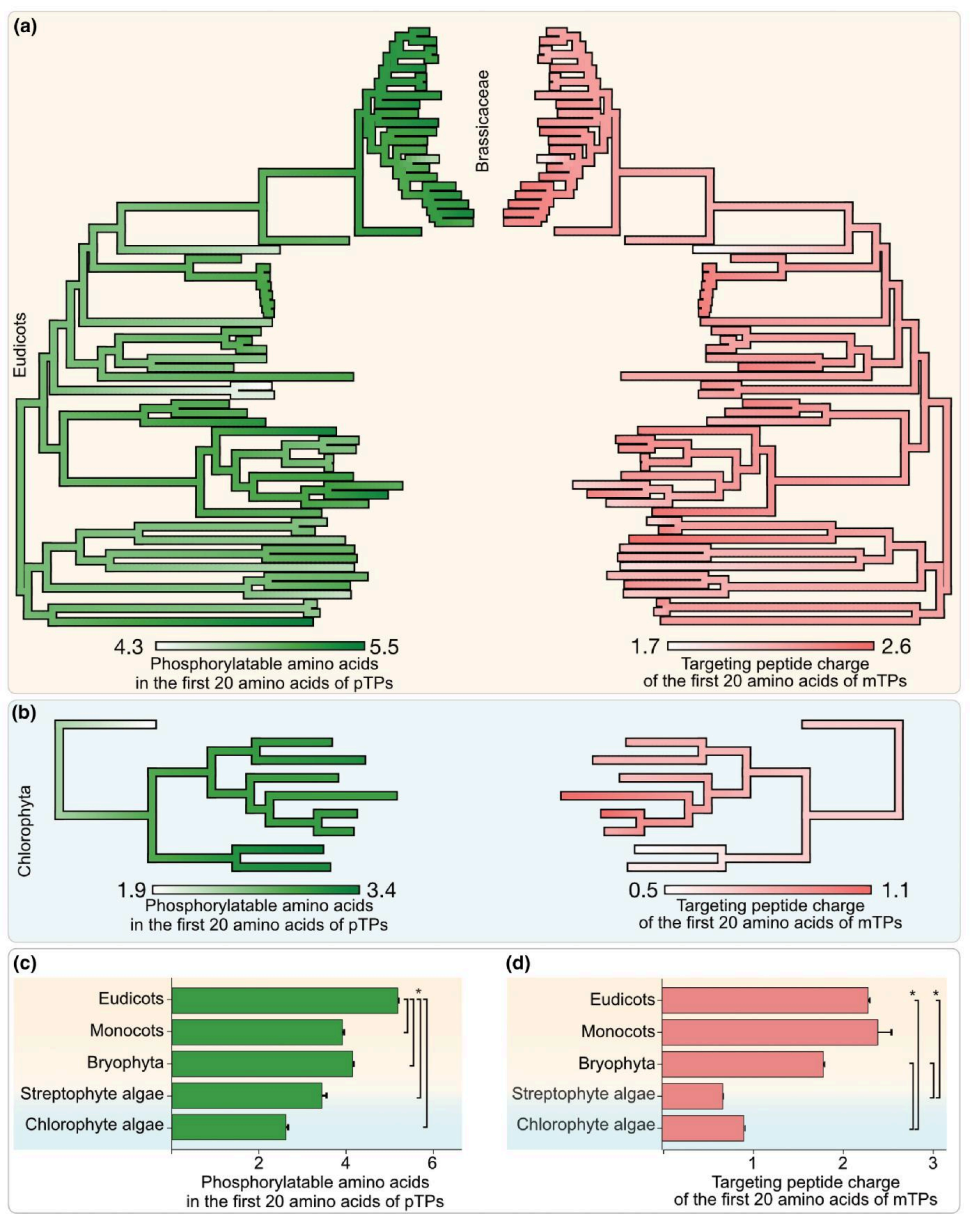
Ancestral state reconstruction of TP divergence. Ancestral states of phosphorylatable AA in the first 20 AA of pTPs and charges of mTPs for five plant lineages were reconstructed using phylogenies for each lineage (Fig. S2) and the phylogenies were color-coded based on these values across ancestors. A complete phylogeny and ancestor state values are shown for a) eudicots and b) chlorophyte algae. Based on a similar reconstruction, inferred means of phosphorylatable AA residues across c) all pTPs and means of positive charges across and d) all mTPs of the last common ancestors of each group are plotted (c to d, *P < 0.05, Welch's *t*-test). The complete ASR of streptophyte algae, bryophytes and monocots, together with the raw data for all five clades, is summarized in Fig. S3 and its source data.

To investigate where organelle TPs began to diverge during evolution (e.g. at the base of chloroplastida vs. later along with the divergence of land plant clades), we reconstructed the ancestral states of the two traits of interest (mean charges and phosphorylatable AA) at the beginning of the TPs across five plant lineages (Fig. 2, Figs S2 to S3) using these trait values in extant species (Fig. 1). A comparison between eudicots and chlorophyte algae ancestors underscores that at the eudicot ancestral node, the pTPs were already enriched in phosphorylatable AA and the mTPs accumulated positively charged AA, traits that were much less prominent in the chlorophyte ancestor (Fig. 2). Among eudicots, the mustard and brassicaceae families showed that the highest values and changes in the two traits occurred concurrently: species with a higher positive charge in mTPs also showed a higher number of phosphorylatable AA in the pTPs, and vice versa. The concomitant divergence of both is likely to aid in further import specificity. On the contrary, in chlorophyte algae, changes in these two traits did not occur simultaneously, and across species, the variation is high (Fig. 2b). Generally, positive charges in mTPs and phosphorylatable AA in pTPs started to become evident at the time of streptophyte and land plant origin (Fig. 2c and d).

If TP divergence is indeed aiding the correct targeting and maintenance of organelle biology, it is expected to be enriched based on protein function and a contribution to defining the organelle's biology. To probe this, we analyzed a more reliable localization and functional annotation dataset of *Arabidopsis thaliana.* We found that plastid proteins involved in photosystem biology and redox reactions are more enriched in phosphorylatable AA (Fig. S4). They have a lower positive charge than the rest of plastid proteins and of mTPs, i.e. the pTPs of these functional categories are more diverged from the mTPs than generally observed. Some key mitochondrial proteins, e.g. TCA cycle proteins, also show a higher mTP charge than the average of all mTPs (Fig. S4).

### The Divergence of Targeting Sequences Enhances the Accuracy of Protein Sorting

**Fig. 3.**
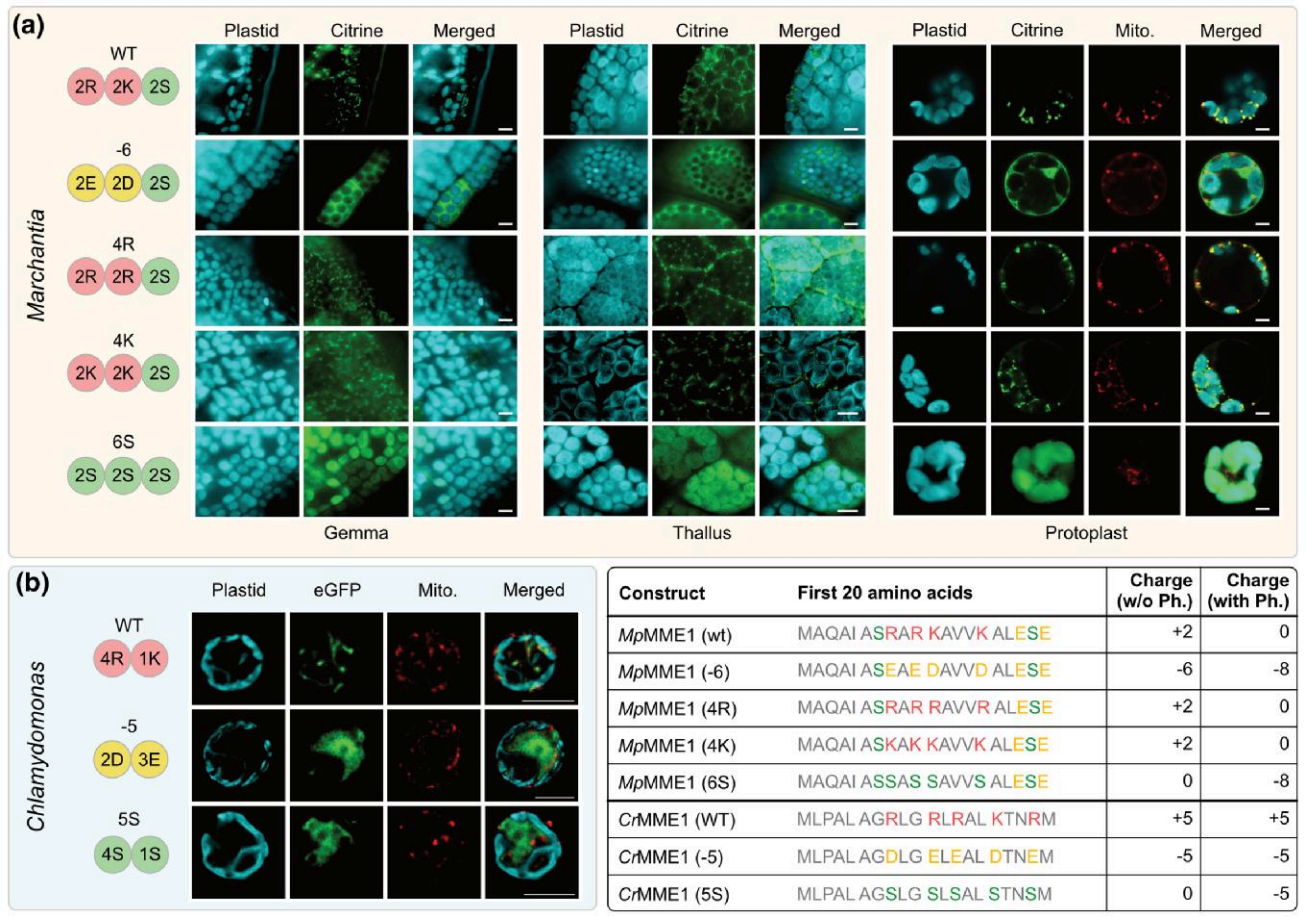
Altering only the charge of the first 20 AA changes the targeting behavior of TPs. Subcellular localization of MME1 homologs in a) the bryophyte plant *Marchantia* and b) the chlorophyte alga *Chlamydomonas*. Each row shows the localization of an independent reporter fusion, and the top row (for each species) represents the wild type (WT). Below the WT are the variants where key AA in the first 20 amino acid region of the TPs were substituted. The numbers of these key AA (in single-letter codes) are shown on the left. The full sequence of the first 20 AA and resulting charge (Ph., phosphorylation) are shown in the table on the bottom right. MME1 (and its variants) was localized in *Marchantia* gemma, thallus, and protoplasts via citrine and in *Chlamydomonas* single cells via eGFP fusion constructs. Plastids were imaged through their autofluorescence and mitochondria through MitoTracker Red. *Chlamydomonas* images were deconvoluted from the Z-stacks and *Marchantia* images are from single plane imaging. Scale bars 5 µm. See Fig. S5 for co-localization analyses and Table S7 for a range of brightness-contrast settings for the images.

To test the hypothesis that the divergence of TP charge alone changes targeting destination, we utilized the mitochondrial malic enzyme 1 (MME1) as an exemplary protein since the first 20 AA of this protein showed a substantial positive charge in algae *Chlamydomonas reinhardtii* and *Marchantia polymorpha* (a system to study land plant evolution [Bowman et al. 2017; Bowman et al. 2022]), allowing us to lower the charge and generate variants in both algae and land plant. Cloning of the MME1 homolog of *C. reinhardtii* (*Cr*MME1; Cre06.g268750) proved difficult, but the first 20 AA of the mitochondrial protein, with a charge of +5, are sufficient for correct mitochondrial targeting and the positive charge in the TP is crucial, as constructs in which the charge is changed to −5 or 0 localize predominantly in the cytosol (Fig. 3, Fig. S5). The MME1 homolog of *M. polymorpha* (*Mp*MME1; Mp4g02270) has an overall charge of +4. The two arginine and two lysine residues that make up the positive charge allowed us to address the relative contributions of R vs. K. As predicted, the reporter fusion with the full *Mp*MME1 sequence shows a mitochondrial localization, while the −4 mutant construct accumulates in the cytosol (Fig. 3). It is debated, whether the positive charge or AA identity per se is crucial for mitochondrial import (Lee et al. 2019; Caspari et al. 2023). Substituting arginine for lysine (and vice versa) still leads to correct mitochondrial targeting (Fig. 3), and so for *Mp*MME1, a positive charge matters and not whether it is mediated by arginine or lysine. The analysis of the first 20 AA of 214 mTPs from 72 eudicots, where the positive charge is pronounced (above +3), shows a general preference for arginine (Fig. S6). Those proteins that almost exclusively use arginine in the mTPs are bioenergetics related, while those that strongly prefer lysine are mostly information processing related (Fig. S6). A preference for either lysine or arginine might hence depend on the context of protein function, which appears odd considering targeting sequences are cleaved upon import into the matrix (Ghifari et al. 2019).

Regardless of AA identity, the divergence between mitochondrial and plastid TPs (and an enrichment of serines in pTPs) appears to be a necessity. When the positively charged AA of the *Mp*MME1 mTP are substituted with serine residues, the fusion construct now predominantly targets the plastids. Only in the protoplast preparations did we observe some targeting of the construct to mitochondria (Fig. 3a, Fig. S5). It shows that replacing positively charged AA with serine residues is sufficient to re-direct—or, in this case, mistarget—one of the key mitochondrial marker proteins from mitochondria to plastids. Note that a similar experiment in *Chlamydomonas* did not result in any targeting of *Cr*MME1 to the plastid (Fig. 3). This is maybe because of a requirement, of unknown nature, for a generally longer pTP in the chlorophyte alga (Caspari 2022) and a possible lack of phosphorylation in light of an evolutionary late enrichment of phosphorylatable AA in pTPs (Figs 2 and 3). These observations, together with a previous study showing mistargeting of rbcS to mitochondria upon serine-to-arginine substitutions (generating a chloroplast-aversion signal) (Lee et al. 2019), underscore the importance of the initial 20 AA in determining organelle specificity.

### Origin of a Membrane-anchored TP Dephosphorylase at the Water-to-Land Transition

**Fig. 4.**
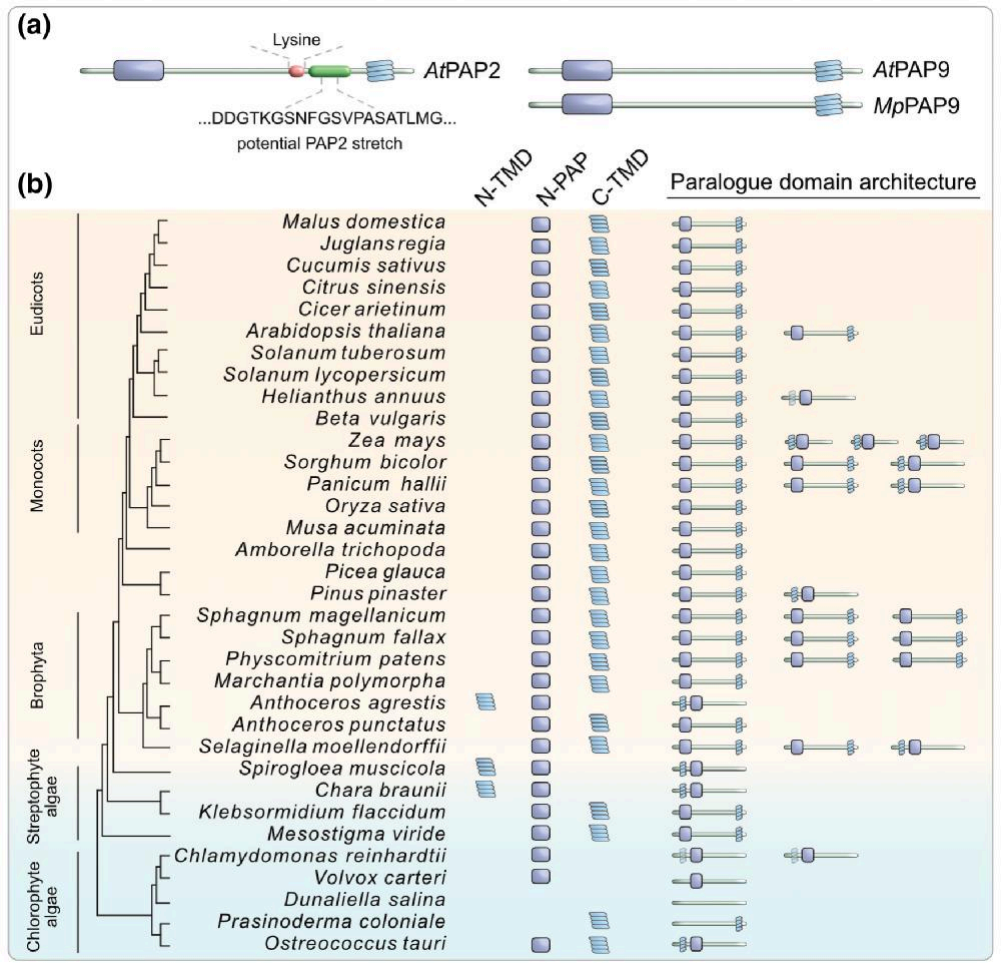
Membrane-docking of PAP2/9 evolved at the time of plant terrestrialization. a) Key differences between *Arabidopsis* PAP2 and PAP9 include an additional stretch of AA before the C-terminal membrane domain and a lysine couplet present in PAP2. The *Marchantia* PAP2/9 homolog lacks these features and is likely a PAP9. b) Domain architecture of the PAP2/9 family across various Chloroplastida. A characteristic N-terminal PAP domain (N-PAP) already evolved in chlorophyte algae, but a C-tail membrane anchor only later in streptophytes. Copy number increase in certain species is indicated by the additional icons under the column paralog domain architecture.

Two purple acid phosphatases, PAP2 and PAP9, are C-tail anchored into the outer plastid membrane in *Arabidopsis*, where they work on pTPs (Sun et al. 2012; Zhang et al. 2016; Zhang et al. 2021). *At*PAP2 (AT1G13900) and *At*PAP9 (AT2G03450) share a 70% global sequence identity and are both characterized by the presence of an N-terminal purple acid phosphatase domain (N-PAP) and a C-tail anchor (C-TMD; Fig. 4). In addition, *At*PAP2 contains two lysine residues and a stretch of AA preceding the C-TMD, which, unlike PAP9, might enable *At*PAP2 to simultaneously anchor into the mitochondrial outer membrane (Zhang et al. 2021) to also process mTPs (Law et al. 2015).

Purple acid phosphatases are highly conserved across all domains of life (Bhadouria and Giri 2022). We clustered all homologs gene families from 42 species (Table S4c) based on sequence similarity (Emms and Kelly 2019) and screened the clusters (Table S4b) for known import components (Table S4c-d). Our protein clustering identified *At*PAP2 and *At*PAP9 to be part of a smaller and likely Chloroplastida-specific family of PAPs that we termed the PAP2/9 family. Like many other organelle related proteins, the PAP2/9 family also underwent a substantial copy number increase in embryophytes (Fig. 4). Further sequence analysis suggests that sometime during plant terrestrialization, and commencing in streptophyte algae such as *Klebsormidium*, some of these PAP2/9 members acquired a C-TMD (Fig. 4). In some embryophytes, the members with a C-TMD duplicated and diverged further, likely giving rise to a plastid-anchored PAP9 and a dual-anchored PAP2. To experimentally probe this, we focused on PAP2/9 homologs of *Chlamydomonas* (Cre11.g468500) and *Marchantia* (Mapoly0122s0035). The algal homolog encodes a putative transmembrane domain at the very N-terminus (ca. 1 to 30 AA), whereas the *Marchantia* homolog encodes a land plant-like C-TMD (Fig. S7), albeit lacking an AA stretch before the C-TMD that is present in *At*PAP2. Reporter fusion of the algal N-terminal region alone led to a mitochondrial (likely matrix) localization, whereas that of *Marchantia* localized to the plastid (Fig. S7).

### Remodeling of Import Platforms Alongside Plant Diversification

The protein clustering (Table S4) allowed us to trace the evolutionary history of import components across the Chloroplastida, which shows that many components of mitochondrial and plastid protein import were duplicated around the time of plant terrestrialization (Table S4e, Fig. S8), i.e. from the streptophyte algae *Chara* and *Spirogloea* onwards. This is in line with a more general pattern regarding the expansion of mitochondrial and plastidal functions during the transition from water to land (Raval et al. 2024).

**Fig. 5.**
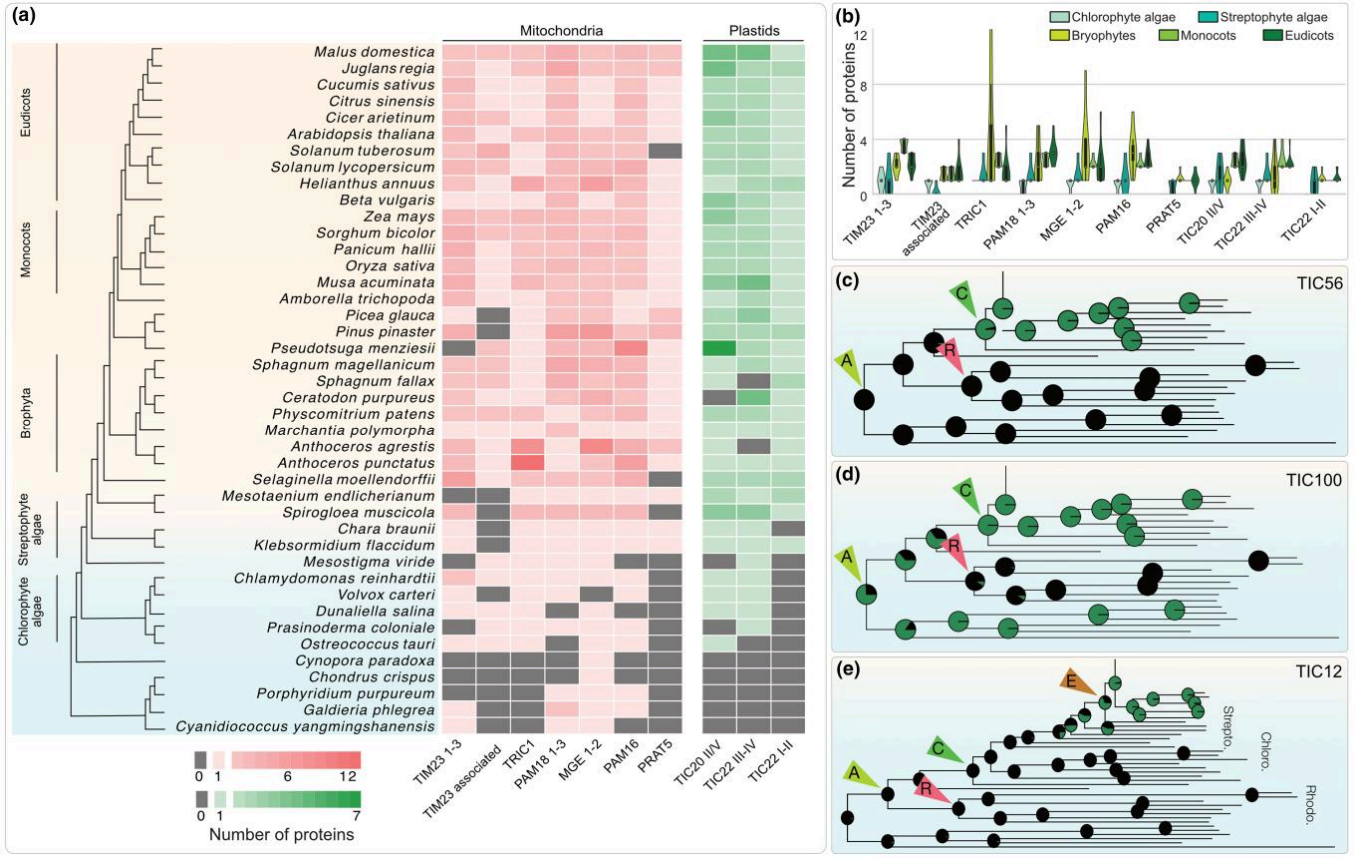
Recruitment, expansion, and divergence of import receptors. a) GCNs of key import channels at the inner membranes of mitochondria (crimson) and plastids (green) across species of interest (*n* = 42 Archaeplastida species) and b) a distribution across each clade of interest. (c to e) Ancestral state reconstruction for the key components of the TIC complex (*n* = 137 Archaeplastida species, six outgroups). Ancestors of Archaeplastida, rhodophytes, chlorophytes, and embryophytes are indicated by light green, red, green, and mustard arrows. The phylogeny of the species used and full ASR of the three import components is shown in Figs S9 to S12.

At the inner mitochondrial membrane, we traced the duplication and divergence of the main import channel TIM23 (Fig. 5a and b). It expanded the TIM23 1 to 3 family and added isoforms such as TIM23-II, which in *Arabidopsis* allows the fine-tuning of protein import in response to changing levels of respiratory complex I occurring, e.g. under oxidative stress (Wang et al. 2012 ). Associated proteins such as TIM21 were also duplicated (Fig. S8), with the copies likely interacting with proteins of the electron transport chain (Murcha et al. 2014b). We also capture duplications of peripheral components of TIM23, TIM22, and the TIM23-17 complex at large, such as PRAT (Preprotein and AA transporters) in the green lineage (Fig. 5a and b, Fig. S8). These newly recruited components, which connect protein import with respiration and oxidative stress, likely benefited a life on land where oxidative stress is more pronounced. At the outer mitochondrial membrane, the receptors TOM20 and TOM22, and the main import channel TOM40, also significantly diverged from their nonphotosynthetic homologs (Duncan et al. 2013). One reason could be the need for better cargo selection now that the plastid endosymbiont was present with its own emerging import apparatus, namely TOC/TIC (Garg and Gould 2016). That is in accordance with the *Arabidopsis* mitochondrial TPs, unlike those of animals, displaying two hydrophobic domains that bind to two separate binding sites of the *At*TOM20 (Abe et al. 2000; Perry et al. 2006; Rimmer et al. 2011; Murcha et al. 2014a; Murcha et al. 2014c; Heidorn-Czarna et al. 2022 ). In combination, the changes at the two mitochondrial membranes likely improved import rate, accuracy, and regulation.

At the inner plastid membrane, duplications of TIC20, TIC62, and TIC22 (Fig. 5, Fig. S8) gave rise to quantitative and qualitative differences in plastid import. For instance, a duplication of TIC22, captured as two TIC22 sub-families (Fig. 5a), provided functionally redundant isoforms that increase the rate of import in land plants (Rudolf et al. 2013). In contrast, isoforms resulting from duplications of TIC20 contributed to a qualitatively different import complex characterized by TIC20, TIC56, and TIC214 (Kikuchi et al. 2013). While the green lineage-specific TIC20-IV (Fig. 5a and b) and TIC56 (Fig. 5c) and an ancient TIC100 (Fig. 5d) formed the core of this complex, a massive divergence of another TIC20 isoform likely resulted in TIC214, which only shares homology to TIC20 in the region encoding the six transmembrane spanning domains (de Vries et al. 2015). After its emergence in Chloroplastida, as also substantiated by its presence in *Chlamydomonas* (Jin et al. 2022; Liu et al. 2023), this complex was further modified in land plants via the addition of TIC12 (Fig. 5d), a recently identified component of this complex (Zhao et al. 2022 ).

## Discussion

The eukaryotic cell is required to orchestrate the correct targeting of thousands of cytosolically translated proteins. Eukaryotes, especially photosynthetic ones, have therefore evolved a variety of mechanisms that contribute to the correct targeting and import selection: organellar mRNA localization (Weis et al. 2013; Tian and Okita 2014), alternative transcription and translation initiation (Mackenzie 2005), ubiquitination (Thomson et al. 2020), piggy-back transport (Thoms 2015), phosphorylation (May and Soll 2000; Garg and Gould 2016; Lee et al. 2023), and a plethora of small GTPases when taking vesicle trafficking as a mean to specifically transport proteins from one destination to another into account (Stenmark 2009; Guo et al. 2014). Some work constitutively, while others secure protein homeostasis of a compartment and can swiftly respond to environmental changes (Bauer et al. 2015). Each import strategy adapts according to the requirements of a specific evolutionary niche and, conversely, changes in protein targeting can offer novel possibilities.

**Fig. 6.**
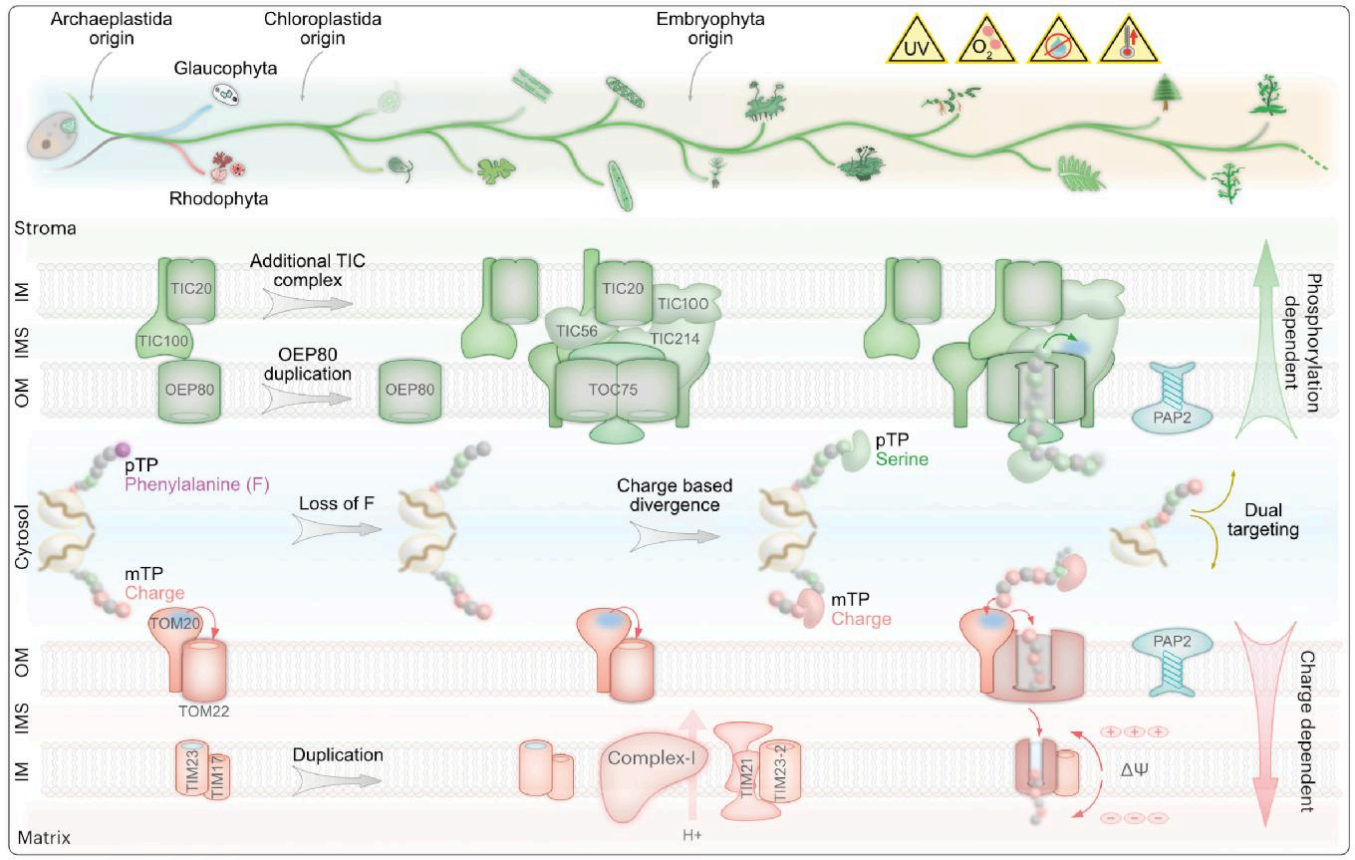
Schematic of mitochondrial and plastid protein import evolution. For simplicity, a linear evolution of the main archaeplastidal lineages is pictured at the top. Chloroplastidal diversification on land was characterized by organellar adaptation toward various stressors (Raval et al. 2024) and a recruitment of novel proteins that require import. At the origin of the Archaeplastida, a phenylalanine-based motif in the pTP aided the plastid targeting as the first point of discrimination between mitochondria and plastids. That feature was lost at the origin of the green (chloroplastidal) lineage, arguably also lowering the import specificity and causing some protein mistargeting between plastids and mitochondria (Knopp et al. 2020). The selection against such a mistranslation at the origin of the green lineage and subsequently during its evolution (in light of the expansion of the organelle proteomes in the land plants [Raval et al. 2024]), contributed to the evolutionary remodeling of the import machinery. Duplication and divergence of key import channels (e.g. TOC75, TIC20, and TIM23) gave rise to components such as an additional, TIC214-based import complex. It aided in the import of an expanded organelle proteome, and likely at an increased rate, facilitating adaptation to light and oxidative stress (Knopp et al. 2020). On the cargo side, the N-terminal targeting sequences for plastids (pTPs) and mitochondria (mTPs) diverged, whereby pTPs accumulated phosphorylatable AA (depicted in green) and averted positively charged AA (depicted in Salmon) and hereby added a component from which to differentiate import cargo in a phosphorylation- and charge-dependent manner. The evolutionary divergence of TPs was undergirded by Δψ and a coevolution of receptors by mechanisms such as (i) initial, and likely ancient, charge-based screening by the outer membrane receptor TOM20/44, and (ii) a Δψ-dependent activation of a cation-selective channel TIM23, screening for positively charged mTPs on the matrix side. Chaperons might improve accuracy (May and Soll 2000; Qbadou et al. 2006; Jarvis 2008; Lamberti et al. 2011) and the membrane recruitment of a dephosphorylase such as PAP2 likely contributed to import the regulation of (dual-targeted) cargo.

The expansion of plastid proteomes underpinned the evolutionary success of the chloroplastidal lineage and its subsequent adaptation to the land and its novel stressors (de Vries et al. 2016; Raval et al. 2024). Concomitant changes of the import machinery, such as a duplication of the main outer membrane import channel TOC75, allowed for a better rate of import of an increasing amount of cargo, which, in return, is likely associated, for instance, with a better response to high light stress in the green lineage (Knopp et al. 2020). Similarly, the expansions of plastidal and mitochondrial inner membrane channel components (Fig. 6, Fig. S8) likely increased import rate, which can help to adapt to altering levels of oxidative stress by better regulating import.

Co-evolution of import receptors and N-terminal targeting sequences also contributed to improved cargo selection. For instance, TOM20 and TOM22, which mediate the entry of the mTP (Rimmer et al. 2011), appear to be substantially diverged in Archaeplastida, and the mechanism of cargo selection differs between plants and animals: in animals, the cytosolic domain of TOM22 interacts with the mTP and imports it further, while the plant TOM22 serves as a gate-keeper by occupying a hydrophobic cradle on TOM20 (Macasev et al. 2000; Carrie et al. 2010; Rimmer et al. 2011). The plant TOM22 gets outcompeted by mTPs and displaced, but likely not by a pTP (Macasev et al. 2000; Carrie et al. 2010; Rimmer et al. 2011), which adds the kind of TP divergence we observe for angiosperms but not algae (Figs 1 and 2). TOM22 interacts with the mTPs via a negatively charged region and likely selects positively charged mTPs in the glaucophyte alga *Cyanidioschyzon* (Hirata et al. 2024), once more underscoring the relevance of charge-based selection for organelle cargo at the outer mitochondrial membrane in early branching phototrophic eukaryotes. Ancestral states of these groups suggest that the divergence of charge predates the diversification within eudicots and angiosperms, and it might have played a role in the evolutionary success of the groups.

At the inner mitochondrial membrane, TIM23 forms a cation-selective channel that is activated by the mTP and the membrane potential (Δψ) of the membrane (Truscott et al. 2001). Δψ can actively facilitate the entry of positively charged mTPs, while averting a negatively charged one, such as a phosphorylated pTP (Garg and Gould 2016). At least two major receptors at the inner and outer membranes, along with Δψ, hence contribute to a selection pressure that suppresses positive charges in pTPs. It is this divergence that our substitutions erase, resulting in the mis-targeting (and dual targeting in the case of protoplasts) of a mitochondrial marker protein (Fig. 3).

On the plastid side, changes at the inner envelope membrane and in the pTP also improved the accuracy of protein import. It is feasible that the chloroplastidal recruitment of the TIC214-containing import complex (Fig. 5) further aided in improving the level of import accuracy that was at least partly compromised with the loss of a phenylalanine-based TP selection system at the origin of the Chloroplastida (Knopp et al. 2020). Molecular dynamics simulation on the TIC–TOC super complex structure predicted an interaction of the pTP with TIC214 (Liu et al. 2023), which was confirmed by cross-linking experiments that revealed an interaction of the rbcS TP with positively charged residues of TIC214 in its preprotein translocation path (Jin et al. 2022). Such an interaction provides a possibility to differentiate better between the TPs (e.g. repel the generally positively charged mTP and select a pTP), contributing to the evolutionary divergence of the TPs witnessed along with the late diversification of plants.

Evolving phosphorylatable sites in a pTS only serve a purpose when accompanied by appropriate kinases and phosphatases. The kinase STY17 in *Arabidopsis* phosphorylates pTPs, which is then imported at an increased rate, aided by HSP70–14/3/3 guidance complex (Waegemann and Rgen Soll 1996; May and Soll 2000; Martin et al. 2006; Lamberti et al. 2011; Schwenkert et al. 2011; Von Loeffelholz et al. 2011; Law et al. 2018). The evolutionary history of such chaperons, kinases, and phosphatases remains blurry, owing to the conserved nature of functional domains and their general abundance. By combining protein clustering, domain analyses, and experimental approaches, however, we were able to verify the embryophytic recruitment of at least one outer plastid membrane-localized dephosphorylase, a member of the PAP2/9 family (Fig. 4), which plays a role in protein import in *Arabidopsis* (Law et al. 2015; Zhang et al. 2016). Identifying the members of this large protein family in individual species remains challenging and notwithstanding the caveats common to orthologue annotations, we identify homologs of *At*PAP2 in *Chlamydomonas* and *Marchantia*. The mitochondrial localization of *Cr*PAP2 might hint at the ancestral localization of the PAP2/9 protein family represented by chlorophyte algae. *Marchantia* encodes only a single C-TMD containing PAP (Mapoly0122s0035), which lacks the characteristic AA stretch present in *At*PAP2 but encodes a C-TMD (Fig. S7). We refer to this protein as *Mp*PAP9, whose plastid localization (Fig. S7) might capture an intermediate state of PAP2/PAP9 protein family evolution. Along with a concomitant enrichment of phosphorylatable AA in the pTPs, particularly pronounced in angiosperms (Figs 1 and 2), a recruitment of a plastid outer membrane dephosphorylase (Fig. 4) hints at a layer of differential targeting control to mitochondria and plastids still poorly understood (Fig. 6).

A duplication of this membrane-anchored phosphatase, likely in angiosperms (Fig. 4), gave rise to a paralog such as *At*PAP2, which is dually localized to plastid and mitochondrial outer membranes (Sun et al. 2012; Zhang et al. 2016; Zhang et al. 2021). Since phosphorylated AA in the pTPs are thought to act as a mitochondria aversion signal (Lee et al. 2006; Garg and Gould 2016), the presence of a dephosphorylase on the mitochondrial outer membrane appears to functionally reverse the evolutionary changes, potentially allowing for organelle mis- or dual-targeting. Indeed, a known substrate of *At*PAP2 is a dTP, mitochondrial import of which hinges upon de/phosphorylation (Law et al. 2015). Some 110 proteins in *Arabidopsis* are known to be dually targeted (Carrie et al. 2009; Carrie and Small 2013), and the dTPs physicochemical features represent an intermediate value between those of pTPs and mTPs (Garg and Gould 2016).

The relative contributions of TP secondary structure and its physicochemical features to correct targeting are a matter of ongoing investigations, especially due to a lack of distinct features other than the abundance of serines in pTPs and alpha helices in mTPs (von HEIJNE et al. 1989; Bruce 2000; Jarvis 2008; Sidorczuk et al. 2023; Thagun et al. 2024). Structural predictions for the wild-type MME1 sequences from the *Chlamydomonas* and *Marchantia* (predicted using Alfafold3 (Abramson et al. 2024; Fig. S13) show them to have an alpha helix in the first 20 AA. The *Marchantia* MME1 with six serines and an intact alpha helix, however, indeed localizes to the plastid and provides an example, where physicochemical features surpass the secondary structure to convert a mitochondrial protein into a dual-targeted one. To what extent this could be a way toward dual targeting remains to be addressed, along with the role of de/phosphorylation (e.g. by PAP2), and the mechanism for the membrane recruitment of dephosphorylases. That the first 20 AA of *Cr*MME1 are sufficient for correct mitochondrial targeting is in line with an evolutionary early dependence of positive charges for mitochondrial matrix targeting, which manifested itself during eukaryogenesis and in early diverging eukaryotes (Garg et al. 2015; Gould et al. 2016; Hirata et al. 2024). It was a necessity before plastid origin and pTPs evolved accordingly (Knopp et al. 2020) (Fig. 6).

A subset of plastid proteins, including the proteins involved in metabolism and translation, have a pTP charge closer to the mean mTP charge, i.e. the pTP of these proteins are less diverged from mTPs. Moreover, proteins involved in translation in general have a wider distribution of charge and number of phosphorylatable AA. Proteins whose mistargeting is detrimental are likely under stronger selection to diverge from mTPs than those whose mistargeting is not. The latter, over time, provides ground to evolve functionally relevant dual targeting. We propose that the gradual loss of phenylalanine-based targeting at the origin of Chloroplastida offered additional ground for the origin of dTPs due to some mis-sorting, and with which import receptors and components, such as PAP2/9, coevolved for an additional layer of import regulation. Experimental information regarding protein dual targeting outside of angiosperms, however, is rare, and the prediction of dTPs remains unreliable (Gould et al. 2024). Studying the origin and evolution of (regulated) protein dual targeting is hence a promising avenue and calls for more dedicated research outside of angiosperms.

In closing, our computational analyses across a wide range of photosynthetic eukaryotes underscore major shifts in the TPs from algae to angiosperms. The sample size of reliable genomes and organelle proteomes is skewed toward embryophytes and affects our inference as to where along the evolution of plants the TPs diverged exactly. Nevertheless, subsampling at the lowest range of organelle protein numbers (*n* = 100 to 300) and species numbers (*N* = 6 to 9) shows that eudicot pTPs are indeed significantly different from eudicot mTPs and from algal TPs (Fig. S14). Additional genomes, organelle proteomes, and experimental studies on protein import in algae (including rhodophyte and glaucophyte), will aid in tracing the evolution of TPs at a finer resolution. The role of TP modifications other than phosphorylation (Giglione and Meinnel 2021; Hoernstein et al. 2024) also remains to be explored. Notwithstanding the current limitations, it is clear that the divergence of physicochemical properties of TPs played a crucial role in preventing protein mistargeting (Figs 2 to 4) and it co-evolved with their processing counterparts at the outer membrane (Fig. 5). In light of the loss of a phenylalanine-based motif from the pTP (Knopp et al. 2020) and a general lack of conserved determinants between the two TPs (von HEIJNE et al. 1989; Jarvis 2008), this divergence improved the initial recognition of cargo at the outer membranes of organelles of endosymbiotic origin (Fig. 6) and enhanced the import accuracy of the expanding organelle proteomes that coincided with the diversification of plants (Raval et al. 2024). We indeed find the divergence between pTP and mTP (across species) to correlate with the total proteome size from algae to angiosperms (Fig. S15). The ecological factors responsible for both, organelle proteome expansion and TP divergence, are likely to include the access to more oxygen and an increase in photosynthesis performance associated with plant terrestrialization (Schreiber et al. 2022).

## Conclusion

Contributions of mitochondria and plastids to plant's adaptation and divergence are plenty (de Vries et al. 2016; de Vries and Gould 2018; Dhabalia Ashok et al. 2024; Raval et al. 2024). In this study, we focused on the evolution of the organelle protein import itself, a gatekeeper for the organellar contributions to species fitness. Origin and diversification of the Chloroplastida, particularly that of embryophytes, was facilitated by major changes in import, also evident in targeting sequence divergence between mitochondrial and plastid cargo. Experimentally contrasting organelle protein import in the single cell alga *C. reinhardtii* with that in the early diverging bryophyte *M. polymorpha*, we validate the importance of targeting sequence divergence with respect to charge for enhancing targeting accuracy. Our revised model highlights novel mechanisms that determine import fidelity and regulation, such as de/phosphorylation at the outer membranes and ΔΨ at the inner mitochondrial membrane. These mechanisms are not confined to plants but also work on mitochondrial-nuclear localization shifts in humans, where the phosphorylation of a transcription factor was recently shown to be responsible for maternal inheritance of mitochondrial DNA (Lee et al. 2023). Membranes, ΔΨ and phosphorylation are traits as old as life itself. How their interplay has evolved into a selection and regulation mechanism for protein cargo transfer across membranes of endosymbiotic origin presents a promising avenue of future research.

## Materials and Methods

### Genomes Used in the Study

To study the evolution of TPs and import components, an in-house database of 137 Archaeplastida and 6 sister species was consolidated from available databases (species names and sources summarized in Table S1a). TPs analyses were conducted on 102 genomes from major clades where organelle proteomes could be reliably inferred based on available experimental proteomes (Table S1b). The presence–absence patterns of import components were plotted for a subset of genomes (Table S1c), representative of each clade, to visualize the broad patterns clearly. The ancestral state reconstructions (ASR) of import components were conducted on all genomes.

### Analyses of TPs from Algae to Angiosperms

To study the features of TPs across organelle-targeted proteins, we used 102 Chloroplastida species from five major clades: Eudicots (66), Monocots (14), Bryophytes (7), Streptophyte (6) and Chlorophytes (9) algae (Table S1b). Within-clade all vs all reciprocal best blasts hits (rbbh) were retrieved for all proteins from all the species of these clades. To infer organelle proteomes of all species in a given clade, we used experimental plastid and mitochondrial protein (Gene IDs of the experimental proteins summarized in Table S2a-c) available across Chloroplastida (Sun et al. 2009; Terashima et al. 2011; Mueller et al. 2014). For a protein in a given species, if an rbbh was found against an experimentally validated organelle protein in the same clade, we inferred that protein to be organelle localized for that species (Gene IDs of inferred proteins summarized in Table S3, method schematic in Fig. S1). As there are no experimental proteomes available for streptophyte algae, we inferred their organelle proteomes based on chlorophyte algae. From these inferred plastid, mitochondrial- and dual-targeted proteomes, we analyzed the first 20 AA for electric charge and content of phosphorylatable AA of each protein using (the script). The resulting 20 AA sequences across species, charge/phospho values of each protein across all species are available on Zendeo; means for all species are consolidated in Source Data Fig. 1 and plotted in Fig. 1.

To investigate ancestral states of these two traits, a phylogenetic tree of each clade was constructed using the concatenation of organelle proteins present in all species in that clade (sequences and Newick files available on Zenedo), in IQ-TREE (Minh et al. 2020) v2.0.3 after aligning them with MAFFT (Katoh and Standley 2013) v7.505. The resulting unrooted trees were rooted using minimal ancestral deviation (Tria et al. 2017). Using this phylogenetic tree for the clade and based on values of charge and phosphorylatable AA across all species in the clade, ASR was conducted in rstudio (Phytool package 0.7.80; Revell 2012) (individual outcomes summarized on Zenedo; means consolidated in Source Data Fig. 2 and plotted in Fig. 2 as well as in Fig. S3 and Source Data Fig. S3).

### Cloning and Transfection

Genes of interest from *Marchantia, MpMME1* (Mp4g02270 alias Mapoly0080s0072) and *MpPAP9* (Mp2g16290 alias Mapoly0122s0035) were amplified from the cDNA and cloned into *Gateway* (Invitrogen) entry vector pDONR221 (Invitrogen) by BP reactions set as per manufacturer's protocol. Substitutions of interest were made into the wild-type MME1 using PCR-mutagenesis. All constructs were transferred at the N-terminal of Citrine on pMpGWB106 (Ishizaki et al. 2015), or at the C-terminal of citrine on pMpGB105 (for *MpPAP9*) via *Gateway* cloning by LR reactions set as per manufacturer's protocol. The N-terminal of interest from *Chlamydomonas CrMME1* (Cre06.g268750) and *CrPAP2* (Cre11.g468500) were in vitro synthesized and amplified from the genomic DNA, respectively. Subsequently, they were cloned at the N-terminal of eGFP into the vector pJR38 (Neupert et al. 2009) by digestion with ndeI–bglII (bglII site was introduced to pJR38 prior to cloning the sequences of interest) and sticky-end ligations.

Localization vectors were integrated into *Marchantia* genome via *Agrobacterium-*mediated transfections previously optimized (Ishizaki et al. 2008). Briefly, the vectors were first introduced into electrocompetent *Agrobacterium tumefaciens* (GV3101 without pSOUP) by electroporation (Bio-Rad GenePulser Xcell, 1.44 kV). Epical notches were cut away from two-week-old thalli and after 2 d of recovery, these explants were incubated with *Agrobacterium* transfectants (starting OD_600_ of 0.02) for 3 d. After washing three to four times with 25 to 30 mL water, the explants were incubated with 1 mg/mL cefotaxime (to remove any leftover Agrobacterium) and again washed two times. The explants were then plated onto Hygromycin (10 μg/mL) and Cefotaxime (100 μg/mL) and selected until they produced gemmae. Gemmae from multiple explants were taken forward to the next generation (G0, the first generation starting from a gemma) and grown until they produced gemmae, which were screened for the reporter expression under the microscope. Expression-positive gemmae were further propagated and used for the localization experiments.

The localization constructs for the alga were introduced to *Chlamydomonas* via the glass bead transfection method (Kindle 1990). Briefly, 1 µg of linearized plasmid, 5% PEG6000, 330 µL *Chlamydomonas* culture (concentrated to 10^8^ cells per ml) and 300 mg glass beads were vortexed for 15 s. The cells were plated, selected, and further propagated on 10 µg/mL paromomycin. The algal and plant lines used in the study are summarized in Table S5.

### Plant and Algal Growth Conditions

*M.* ecotype Takaragaike-1 (Tak-1) plants were grown from gemmae on half-strength Gamborg B5 vitamin agar-plates (GVA) under continuous light (70 μmol m^−2^ s^−1^). Individual colonies of *C. reinhardtii UVM4* (Neupert et al. 2009) were grown in high salt medium supplemented with acetate as a carbon source under continuous light (40 μmol m^−2^ s^−1^).

### Protoplast Isolation

Gemmae from expression-positive plant lines were grown for 1 to 2 weeks on GVA. A 5 to 10 mL of 8% mannitol, 6.3 g/L Gamborg B5 vitamins (MGV) containing 15 mg/ml cellulase and 5 mg/mL macroenzyme R10 was poured onto the plates and incubated overnight with gentle shaking (10 to 20 rpm). The plates were agitated to dislodge the protoplasts, and the liquid was filtered with 50 µm strainer and washed twice with MGV by centrifuging at 300 × *g*, 3 min and gently resuspending by swirling the tube.

### Sample Preparation for Microscopy

*Marchantia* gemmae were placed in 30 µL of water on a slide and covered with a coverslip. *Marchantia* thalli, grown (from gemmae stage) for 2 d, were placed in 30 µL of water on a slide and covered with a coverslip. *Marchantia* protoplasts and *Chlamydomonas* single cells were strained with 300 nM MitoTracker™ Red CMXRos as per manufacturer's instructions (50 µL protoplast or cell suspension in MGV or growth media + 300 nM dye, incubated for 20 min at RT, washed twice by centrifuging the cells at 2,000 × *g* and protoplasts at 100 × *g* for 5 min) to visualize mitochondria and immobilized in 1% soft agarose before microscopic analysis.

### Microscopy

Nikon Eclipse Ti imaging platform (illuminated with Nikon Intensilight C-HGFIE for fluorescence) was connected to the camera via Nikon Digital Sight DS-U3 and to the PC via NIS-Elements Basic Research version 4.30. The light intensity was adjusted via neutral density filters at 16-8 for plastids and 1 for mitochondria (1 = minimum attenuation, maximum light allowed through). Plastids were visualized via chlorophyl autofluorescence at 480/590 nm, GFP/citrine reporters at 470/520 nm, and mitochondria at 540/605. Exposure times were set between 20 and 80 ms for plastids, up to 300 ms for GFP, and 200 to 500 ms for mitochondria (depending upon the extent of staining).

The Z-stacks (0.33 µm per stack) of the whole *Chlamydomonas* cells were imaged and deconvoluted via the Lightning adaptive deconvolution module (Leica Microsystems) on Leica TCS SP8 platform (Leica, Wetzlar, Germany) with an HC PL APO CS2 93× glycerol objective (NA 1.3, Leica), connected to the PC via (LAS-X, Leica). Plastids were visualized via chlorophyl autofluorescence at 510/670 to 710 nm, eGFP reporter at 488/495 to 513, citrine reporter at 510/520 to 540 nm, and mitochondria at 560/580 to 620 (White Light Laser, at 25% strength). Autofluorescence from plastid was filtered out during GFP and mitochondria reading by time gating (emitted signal was collected between 0.8 and 6 ns) (Kodama 2016).

### Microscopy Image Processing

Prior to making the microscopy panels, thresholds of each image were adjusted to filter out the halo/bleeding out signals (retain the middle 50% of the total data by treating each pixel on the plane equally). All colocalization analyses were conducted in ImageJ (Fiji) with the BIOP JACoP plugin (auto thresholds).

### Origin and Evolution of Import Components Across Archaeplastida

In total, 42 Archaeplastida species (Table S4a), from each available representative clade, were clustered using OrthoFinder version 2.5.4 (Emms and Kelly 2019), after all vs all blasts were conducted (*E*-value cutoff 10e−6) using diamond blast version 2.011 (Buchfink et al. 2014). The protein clusters (Table S4b) were annotated based on the presence of experimentally validated import components (Table S4c to d), and gene copy numbers (GCN) for each component were inferred across species (Table S4e), and a subset was plotted in Fig. 5a (see Fig. S8 for GCN plots of all components). PAP2/9 homologs from all species were retrieved from the same clusters that were analyzed through Interproscan (Paysan-Lafosse et al. 2022) and DeepTMHMM (Hallgren et al. 2022) to annotate functional domains and membrane anchoring domains, respectively.

To investigate the origins of a select component more rigorously, we utilized the full database of 137 Archaeaplastida species with 6 outgroup species (Table S1a). Considering the sequence divergence of these components, iterative hidden Markov searches were conducted using HMMER (Eddy 1998) 3.3.2 and seed sequences from Arabidopsis. The homologs thus retrieved were used for ASR in rstudio (Phytool package 0.7.80 [Revell 2012]).

## Supplementary Material

msaf240_Supplementary_Data

## Data Availability

Supplementary figures and tables are available with this submission. Additional data are available on Zenedo (https://zenodo.org/records/15689690), which includes, but is not limited to, the following supplementary data and in-house scripts: Source Data Figs 1 to 5 Source Data Figs S2 to S15 Table S1 Species name, taxonomy; and source of genomes used in the study Table S2 Gene IDs of experimental proteomes used as seeds to infer organelle proteomes Table S3 Gene IDs of inferred organelle proteomes Table S4 Gene IDs of experimentally validated import components and their inferred GCN Table S5 The algal cell lines and plant lines used in this study Table S6 Brightness-contrast settings for microscopy images in Fig. 3 rbbh.py Conduct all vs all BLAST for each clade Organelle_proteome_inference.py Infer organelle proteomes based on rhhb results and experimental proteomes get TP1-20AA.py Get the first 20 AA of inferred organelle proteins get TP1-20AA_charge_phospho.py Get charge and phosphorylatable AA from the first 20 AA of inferred organelle proteins TP_charge_phospho_plots_stats.py Summaries and plot averages of charge and phosphorylatable AA content across species and clades clade_phylogeny.py Infer phylogenetic trees for all clades charge_phospho_for_ASR.py Prepare the charge and phosphorylatable AA content data for ASR TP_ASR.r Infer ASR for charge and phosphorylatable AA content of each clade Import_ASR.r Infer ASR for import components of interest

## References

[msaf240-B1] Abe Y et al Structural basis of presequence recognition by the mitochondrial protein import receptor Tom20. Cell. 2000:100:551–560. 10.1016/S0092-8674(00)80691-1. 10721992

[msaf240-B2] Abramson J et al Accurate structure prediction of biomolecular interactions with AlphaFold 3. Nature. 2024:630:493–500. 10.1038/s41586-024-07487-w. 38718835 PMC11168924

[msaf240-B3] Archibald JM . Endosymbiosis and eukaryotic cell evolution. Curr Biol. 2015:25:R911–R921. 10.1016/j.cub.2015.07.055. 26439354

[msaf240-B4] Bauer NC, Doetsch PW, Corbett AH. Mechanisms regulating protein localization. Traffic. 2015:16:1039–1061. 10.1111/tra.12310. 26172624

[msaf240-B5] Bhadouria J, Giri J. Purple acid phosphatases: roles in phosphate utilization and new emerging functions. Plant Cell Rep. 2022:41:33–51. 10.1007/s00299-021-02773-7. 34402946

[msaf240-B6] Bhushan S, Kuhn C, Berglund AK, Roth C, Glaser E. The role of the N-terminal domain of chloroplast targeting peptides in organellar protein import and miss-sorting. FEBS Lett. 2006:580:3966–3972. 10.1016/j.febslet.2006.06.018. 16806197

[msaf240-B7] Bowman JL et al Insights into land plant evolution garnered from the *Marchantia polymorpha* genome. Cell. 2017:171:287–304.e15. 10.1016/j.cell.2017.09.030. 28985561

[msaf240-B8] Bowman JL et al The renaissance and enlightenment of Marchantia as a model system. Plant Cell. 2022:34:3512–3542. 10.1093/plcell/koac219. 35976122 PMC9516144

[msaf240-B9] Bruce BD . Chloroplast transit peptides: structure, function and evolution. Trends Cell Biol. 2000:10:440–447. 10.1016/S0962-8924(00)01833-X. 10998602

[msaf240-B10] Buchfink B, Xie C, Huson DH. Fast and sensitive protein alignment using DIAMOND. Nat Methods. 2014:12:59–60. 10.1038/nmeth.3176. 25402007

[msaf240-B11] Carrie C et al Approaches to defining dual-targeted proteins in Arabidopsis. Plant J. 2009:57:1128–1139. 10.1111/j.1365-313X.2008.03745.x. 19036033

[msaf240-B12] Carrie C, Murcha MW, Whelan J. An in silico analysis of the mitochondrial protein import apparatus of plants. BMC Plant Biol. 2010:10:249. 10.1186/1471-2229-10-249. 21078193 PMC3095331

[msaf240-B13] Carrie C, Small I. A reevaluation of dual-targeting of proteins to mitochondria and chloroplasts. Biochim Biophys Acta. 2013:1833:253–259. 10.1016/j.bbamcr.2012.05.029. 22683762

[msaf240-B14] Caspari OD . Transit peptides often require downstream unstructured sequence for efficient chloroplast import in *Chlamydomonas reinhardtii*. Front Plant Sci. 2022:13:825797. 10.3389/fpls.2022.825797. 35646025 PMC9133816

[msaf240-B15] Caspari OD et al Converting antimicrobial into targeting peptides reveals key features governing protein import into mitochondria and chloroplasts. Plant Commun. 2023:4:100555. 10.1016/j.xplc.2023.100555. 36733255 PMC10363480

[msaf240-B16] Cramer WA, Shiver JW, Furbacher PN, Keegstra K. Amphipathic β-sheet domains in chloroplast transit peptides. In: Current research in photosynthesis. Springer; 1990. p. 2705–2708.

[msaf240-B17] Day PM, Potter D, Inoue K. Evolution and targeting of Omp85 homologs in the chloroplast outer envelope membrane. Front Plant Sci. 2014:5:535. 10.3389/fpls.2014.00535. 25352854 PMC4195282

[msaf240-B18] den Brave F, Schulte U, Fakler B, Pfanner N, Becker T. Mitochondrial complexome and import network. Trends Cell Biol. 2024:34:578–594. 10.1016/j.tcb.2023.10.004. 37914576

[msaf240-B19] de Vries J, Archibald JM. Plant evolution: landmarks on the path to terrestrial life. New Phytol. 2018:217:1428–1434. 10.1111/nph.14975. 29318635

[msaf240-B20] de Vries J, Gould SB. The monoplastidic bottleneck in algae and plant evolution. J Cell Sci. 2018:131:1–14. 10.1242/jcs.203414. 28893840

[msaf240-B21] de Vries J, Sousa FL, Bölter B, Soll J, Goulda SB. YCF1: a green TIC? Plant Cell. 2015:27:1827–1833. 10.1105/tpc.114.135541. 25818624 PMC4531346

[msaf240-B22] de Vries J, Stanton A, Archibald JM, Gould SB. Streptophyte terrestrialization in light of plastid evolution. Trends Plant Sci. 2016:21:467–476. 10.1016/j.tplants.2016.01.021. 26895731

[msaf240-B23] Dhabalia Ashok A, De Vries S, Darienko T, Irisarri I, De Vries J. Evolutionary assembly of the plant terrestrialization toolkit from protein domains. Proc R Soc Lond B Biol Sci. 2024:291:20240985. 10.1098/rspb.2024.0985. PMC11289646 39081174

[msaf240-B24] Diederichs KA et al Structural insight into mitochondrial β-barrel outer membrane protein biogenesis. Nat Commun. 2020:11:1–13. 10.1038/s41467-020-17144-1. 32620929 PMC7335169

[msaf240-B25] Duncan O, Murcha MW, Whelan J. Unique components of the plant mitochondrial protein import apparatus. Biochim Biophys Acta Mol Cell Res. 2013:1833:304–313. 10.1016/j.bbamcr.2012.02.015. 22406071

[msaf240-B26] Eberhard S, Finazzi G, Wollman FA. The dynamics of photosynthesis. Annu Rev Genet. 2008:42:463–515. 10.1146/annurev.genet.42.110807.091452. 18983262

[msaf240-B27] Eddy S . HMMER user's guide: biological sequence analysis using prole hidden Markov models. 1998. Available from: http://eddylab.org/software/hmmer/Userguide.pdf.

[msaf240-B28] Emms DM, Kelly S. OrthoFinder: phylogenetic orthology inference for comparative genomics. Genome Biol. 2019:20:1–14. 10.1186/s13059-019-1832-y. 31727128 PMC6857279

[msaf240-B29] Garg S et al Conservation of transit peptide-independent protein import into the mitochondrial and hydrogenosomal matrix. Genome Biol Evol. 2015:7:2716–2726. 10.1093/gbe/evv175. 26338186 PMC4607531

[msaf240-B30] Garg SG, Gould SB. The role of charge in protein targeting evolution. Trends Cell Biol. 2016:26:894–905. 10.1016/j.tcb.2016.07.001. 27524662

[msaf240-B31] Ge C, Spånning E, Glaser E, Wieslander Å. Import determinants of organelle-specific and dual targeting peptides of mitochondria and chloroplasts in Arabidopsis thaliana. Mol Plant. 2014:7:121–136. 10.1093/mp/sst148. 24214895

[msaf240-B32] Ghifari AS, Huang S, Murcha MW. The peptidases involved in plant mitochondrial protein import. J Exp Bot. 2019:70:6005–6018. 10.1093/jxb/erz365. 31738432

[msaf240-B33] Giglione C, Meinnel T. Evolution-driven versatility of N terminal acetylation in photoautotrophs. Trends Plant Sci. 2021:26:375–391. 10.1016/j.tplants.2020.11.012. 33384262

[msaf240-B34] Gould SB et al Protein targeting into the complex plastid of cryptophytes. J Mol Evol. 2006:62:674–681. 10.1007/s00239-005-0099-y. 16752208

[msaf240-B35] Gould SB, Garg SG, Martin WF. Bacterial vesicle secretion and the evolutionary origin of the eukaryotic endomembrane system. Trends Microbiol. 2016:24:525–534. 10.1016/j.tim.2016.03.005. 27040918

[msaf240-B36] Gould SB, Magiera J, García CG, Raval PK. Reliability of plastid and mitochondrial localisation prediction declines rapidly with the evolutionary distance to the training set increasing. PLoS Computational Biology. 2024:11:e1012575. 10.1371/journal.pcbi.1012575. PMC11581415 39527633

[msaf240-B37] Gruber A et al Protein targeting into complex diatom plastids: functional characterisation of a specific targeting motif. Plant Mol Biol. 2007:64:519–530. 10.1007/s11103-007-9171-x. 17484021

[msaf240-B38] Guo Y, Sirkis DW, Schekman R. Protein sorting at the trans-Golgi network. Annu Rev Cell Dev Biol. 2014:30:169–206. 10.1146/annurev-cellbio-100913-013012. 25150009

[msaf240-B39] Hallgren J et al DeepTMHMM predicts alpha and beta transmembrane proteins using deep neural networks. BioRxiv. 2022. 10.1101/2022.04.08.487609.

[msaf240-B40] Hatefi Y . The mitochondrial electron transport and oxidative phosphorylation system. Annu Rev Biochem. 1985:54:1015–1069. 10.1146/annurev.bi.54.070185.005055. 2862839

[msaf240-B41] Heidorn-Czarna M, Maziak A, Janska H. Protein processing in plant mitochondria compared to yeast and mammals. Front Plant Sci. 2022:13:824080. 10.3389/fpls.2022.824080. 35185991 PMC8847149

[msaf240-B42] Heinnickel ML, Grossman AR. The GreenCut: re-evaluation of physiological role of previously studied proteins and potential novel protein functions. Photosynth Res. 2013:116:427–436. 10.1007/s11120-013-9882-6. 23873414

[msaf240-B43] Hewitt V, Alcock F, Lithgow T. Minor modifications and major adaptations: the evolution of molecular machines driving mitochondrial protein import. Biochim Biophys Acta Biomembr. 2011:1808:947–954. 10.1016/j.bbamem.2010.07.019. 20659421

[msaf240-B44] Hirata R, Mogi Y, Takahashi K, Nozaki H, Higashiyama T. Simple prerequisite of presequence for mitochondrial protein import in the unicellular red alga Cyanidioschyzon merolae. Journal of Cell Science. 2024:137:jcs262042. 10.1242/jcs.262042. 38940185 PMC11298712

[msaf240-B45] Hoernstein SNW et al A snapshot of the Physcomitrella N-terminome reveals N-terminal methylation of organellar proteins. Plant Cell Rep. 2024:43:250. 10.1007/s00299-024-03329-1. 39361041 PMC11450134

[msaf240-B46] Holbrook K et al Functional analysis of semi-conserved transit peptide motifs and mechanistic implications in precursor targeting and recognition. Mol Plant. 2016:9:1286–1301. 10.1016/j.molp.2016.06.004. 27378725

[msaf240-B47] Hölzl G, Dörmann P. Chloroplast lipids and their biosynthesis. Annu Rev Plant Biol. 2019:70:51–81. 10.1146/annurev-arplant-050718-100202. 30786236

[msaf240-B48] Ishizaki K et al Development of gateway binary vector series with four different selection markers for the liverwort *Marchantia polymorpha*. PLoS One. 2015:10:e0138876. 10.1371/journal.pone.0138876. 26406247 PMC4583185

[msaf240-B49] Ishizaki K, Chiyoda S, Yamato KT, Kohchi T. Agrobacterium-mediated transformation of the haploid liverwort *Marchantia polymorpha* L., an emerging model for plant biology. Plant Cell Physiol. 2008:49:1084–1091. 10.1093/pcp/pcn085. 18535011

[msaf240-B50] Jarvis P . Targeting of nucleus-encoded proteins to chloroplasts in plants. New Phytol. 2008:179:257–285. 10.1111/j.1469-8137.2008.02452.x. 19086173

[msaf240-B51] Jin Z et al Structure of a TOC-TIC supercomplex spanning two chloroplast envelope membranes. Cell. 2022:185:4788–4800.e13. 10.1016/j.cell.2022.10.030. 36413996

[msaf240-B52] Katoh K, Standley DM. MAFFT multiple sequence alignment software version 7: improvements in performance and usability. Mol Biol Evol. 2013:30:772–780. 10.1093/molbev/mst010. 23329690 PMC3603318

[msaf240-B53] Keeling PJ . The endosymbiotic origin, diversification and fate of plastids. Philos Trans R Soc Lond B Biol Sci. 2010:365:729–748. 10.1098/rstb.2009.0103. 20124341 PMC2817223

[msaf240-B54] Kikuchi S et al 2013. Uncovering the protein translocon at the chloroplast inner envelope membrane. Science. 2013;339:571–574 10.1126/science.1229262. 23372012

[msaf240-B55] Kim DH, Hwang I. Direct targeting of proteins from the cytosol to organelles: the ER versus endosymbiotic organelles. Traffic. 2013:14:613–621. 10.1111/tra.12043. 23331847

[msaf240-B56] Kindle Karen L. High-frequency nuclear transformation of Chlamydomonas reinhardtii. Proceedings of the National Academy of Sciences. 1990:87:1228–1232. 10.1073/pnas.87.3.1228. PMC53444 2105499

[msaf240-B57] Knopp M, Garg SG, Handrich M, Gould SB. Major changes in plastid protein import and the origin of the chloroplastida. iScience. 2020:23:100896. 10.1016/j.isci.2020.100896. 32088393 PMC7038456

[msaf240-B58] Kodama Y . Time gating of chloroplast autofluorescence allows clearer fluorescence imaging in planta. PLoS One. 2016:11:1–8. 10.1371/journal.pone.0152484. PMC4814121 27027881

[msaf240-B59] Köhler D et al Identification of protein N-termini in Cyanophora paradoxa cyanelles: transit peptide composition and sequence determinants for precursor maturation. Front Plant Sci. 2015:6:1–11. 10.3389/fpls.2015.00559. 26257763 PMC4510345

[msaf240-B60] Lamberti G, Gügel IL, Meurer J, Soll J, Schwenkert S. The cytosolic kinases STY8, STY17, and STY46 are involved in chloroplast differentiation in arabidopsis. Plant Physiol. 2011:157:70–85. 10.1104/pp.111.182774. 21799034 PMC3165899

[msaf240-B61] Law YS et al Phosphorylation and dephosphorylation of the presequence of precursor MULTIPLE ORGANELLAR RNA EDITING FACTOR3 during import into mitochondria from Arabidopsis. Plant Physiol. 2015:169:1344–1355. 10.1104/pp.15.01115. 26304849 PMC4587475

[msaf240-B62] Law YS et al Multiple kinases can phosphorylate the N-terminal sequences of mitochondrial proteins in *Arabidopsis thaliana*. Front Plant Sci. 2018:9:1–14. 10.3389/fpls.2018.00982. 30042778 PMC6048449

[msaf240-B63] Lee DW et al Functional characterization of sequence motifs in the transit peptide of Arabidopsis small subunit of rubisco. Plant Physiol. 2006a:140:466–483. 10.1104/pp.105.074575. 16384899 PMC1361317

[msaf240-B64] Lee DW et al Molecular mechanism of the specificity of protein import into chloroplasts and mitochondria in plant cells. Mol Plant. 2019:12:951–966. 10.1016/j.molp.2019.03.003. 30890495

[msaf240-B65] Lee J, O’Neill RC, Park MW, Gravel M, Braun PE. Mitochondrial localization of CNP2 is regulated by phosphorylation of the N-terminal targeting signal by PKC: implications of a mitochondrial function for CNP2 in glial and non-glial cells. Mol Cell Neurosci. 2006b:31:446–462. 10.1016/j.mcn.2005.10.017. 16343930

[msaf240-B66] Lee W et al Molecular basis for maternal inheritance of human mitochondrial DNA. Nat Genet. 2023:55:1632–1639. 10.1038/s41588-023-01505-9. 37723262 PMC10763495

[msaf240-B67] Leebens-Mack JH et al One thousand plant transcriptomes and the phylogenomics of green plants. Nature. 2019:574:679–685. 10.1038/s41586-019-1693-2. 31645766 PMC6872490

[msaf240-B68] Liu H, Li A, Rochaix J-D, Liu Z. Architecture of chloroplast TOC-TIC translocon supercomplex. Nature. 2023:615:349–357. 10.1038/s41586-023-05744-y. 36702157

[msaf240-B69] Macasev D, Newbigin E, Whelan J, Lithgow T. How do plant mitochondria avoid importing chloroplast proteins? Components of the import apparatus Tom20 and Tom22 from Arabidopsis differ from their fungal counterparts. Plant Physiol. 2000:123:811–816. 10.1104/pp.123.3.811. 10889230 PMC1539262

[msaf240-B70] Mackenzie SA . Plant organellar protein targeting: a traffic plan still under construction. Trends Cell Biol. 2005:15:548–554. 10.1016/j.tcb.2005.08.007. 16143534

[msaf240-B71] Martin T et al A protein kinase family in Arabidopsis phosphorylates chloroplast precursor proteins. J Biol Chem. 2006:281:40216–40223. 10.1074/jbc.M606580200. 17090544

[msaf240-B72] Martin WF, Garg S, Zimorski V. Endosymbiotic theories for eukaryote origin. Philos Trans R Soc Lond B Biol Sci. 2015:370:20140330. 10.1098/rstb.2014.0330. 26323761 PMC4571569

[msaf240-B73] May T, Soll J. 14-3-3 proteins form a guidance complex with chloroplast precursor proteins in plants. Plant Cell. 2000:12:53–63. 10.1105/tpc.12.1.53. 10634907 PMC140214

[msaf240-B74] McKinnon L, Theg SM. Determinants of the specificity of protein targeting to chloroplasts or mitochondria. Mol Plant. 2019:12:893–895. 10.1016/j.molp.2019.05.004. 31128277

[msaf240-B75] Mesmin B . Mitochondrial lipid transport and biosynthesis: a complex balance. J Cell Biol. 2016:214:9–11. 10.1083/jcb.201606069. 27354376 PMC4932376

[msaf240-B76] Minh BQ et al IQ-TREE 2: new models and efficient methods for phylogenetic inference in the genomic era. Mol Biol Evol. 2020:37:1530–1534. 10.1093/molbev/msaa015. 32011700 PMC7182206

[msaf240-B77] Mueller SJ et al Quantitative analysis of the mitochondrial and plastid proteomes of the moss *Physcomitrella patens* reveals protein macrocompartmentation and microcompartmentation. Plant Physiol. 2014:164:2081–2095. 10.1104/pp.114.235754. 24515833 PMC3982764

[msaf240-B78] Murcha MW et al Protein import into plant mitochondria: signals, machinery, processing, and regulation. J Exp Bot. 2014a:65:6301–6335. 10.1093/jxb/eru399. 25324401

[msaf240-B79] Murcha MW, Kubiszewski-Jakubiak S, Wang Y, Whelan J. Evidence for interactions between the mitochondrial import apparatus and respiratory chain complexes via Tim21-like proteins in Arabidopsis. Front Plant Sci. 2014b:5:82. 10.3389/fpls.2014.00082. 24653731 PMC3949100

[msaf240-B80] Murcha MW, Wang Y, Narsai R, Whelan J. The plant mitochondrial protein import apparatus—the differences make it interesting. Biochim Biophys Acta Gen Subj. 2014c:1840:1233–1245. 10.1016/j.bbagen.2013.09.026. 24080405

[msaf240-B81] Neupert J, Karcher D, Bock R. Generation of Chlamydomonas strains that efficiently express nuclear transgenes. Plant J. 2009:57:1140–1150. 10.1111/j.1365-313X.2008.03746.x. 19036032

[msaf240-B82] Paysan-Lafosse T et al InterPro in 2022. Nucleic Acids Res. 2022:51:418–427. 10.1093/nar/gkac993. PMC9825450 36350672

[msaf240-B83] Perry AJ, Hulett JM, Likić VA, Lithgow T, Gooley PR. Convergent evolution of receptors for protein import into mitochondria. Curr Biol. 2006:16:221–229. 10.1016/j.cub.2005.12.034. 16461275

[msaf240-B84] Pujol C, Maréchal-Drouard L, Duchêne AM. How can organellar protein N-terminal sequences be dual targeting signals? In silico analysis and mutagenesis approach. J Mol Biol. 2007:369:356–367. 10.1016/j.jmb.2007.03.015. 17433818

[msaf240-B85] Qbadou S et al The molecular chaperone Hsp90 delivers precursor proteins to the chloroplast import receptor Toc64. EMBO J. 2006:25:1836–1847. 10.1038/sj.emboj.7601091. 16619024 PMC1456943

[msaf240-B86] Raval PK, MacLeod AI, Gould SB. A molecular atlas of plastid and mitochondrial proteins reveals organellar remodeling during plant evolutionary transitions from algae to angiosperms. PLoS Biol. 2024:22:e3002608. 10.1371/journal.pbio.3002608. 38713727 PMC11135702

[msaf240-B87] Revell LJ . Phytools: an R package for phylogenetic comparative biology (and other things). Methods Ecol Evol. 2012:3:217–223. 10.1111/j.2041-210X.2011.00169.x.

[msaf240-B88] Richardson LGL, Schnell DJ. Origins, function, and regulation of the TOC-TIC general protein import machinery of plastids. J Exp Bot. 2020:71:1226–1238. 10.1093/jxb/erz517. 31730153 PMC7031061

[msaf240-B89] Rimmer KA et al Recognition of mitochondrial targeting sequences by the import receptors Tom20 and Tom22. J Mol Biol. 2011:405:804–818. 10.1016/j.jmb.2010.11.017. 21087612

[msaf240-B90] Rudolf M et al In vivo function of Tic22, a protein import component of the intermembrane space of chloroplasts. Mol Plant. 2013:6:817–829. 10.1093/mp/sss114. 23204504

[msaf240-B91] Schreiber M, Rensing SA, Gould SB. The greening ashore. Trends Plant Sci. 2022:27:847–857. 10.1016/j.tplants.2022.05.005. 35739050

[msaf240-B92] Schulte U et al Mitochondrial complexome reveals quality-control pathways of protein import. Nature. 2023:614:153–159. 10.1038/s41586-022-05641-w. 36697829 PMC9892010

[msaf240-B93] Schwenkert S, Soll J, Bölter B. Protein import into chloroplasts-how chaperones feature into the game. Biochim Biophys Acta Biomembr. 2011:1808:901–911. 10.1016/j.bbamem.2010.07.021. 20682282

[msaf240-B94] Scotti PA et al Yidc, the *Escherichia coli* homologue of mitochondrial Oxa1p, is a component of the Sec translocase. EMBO J. 2000:19:542. 10.1093/emboj/19.4.542. 10675323 PMC305592

[msaf240-B95] Sidorczuk K, Mackiewicz P, Pietluch F, Gagat P. Characterization of signal and transit peptides based on motif composition and taxon-specific patterns. Sci Rep. 2023:13:15751. 10.1038/s41598-023-42987-1. 37735485 PMC10514287

[msaf240-B96] Steiner JM, Yusa F, Pompe JA, Löffelhardt W. Homologous protein import machineries in chloroplasts and cyanelles. Plant J. 2005:44:646–652. 10.1111/j.1365-313X.2005.02559.x. 16262713

[msaf240-B97] Stenmark H . Rab GTPases as coordinators of vesicle traffic. Nat Rev Mol Cell Biol. 2009:10:513–525. 10.1038/nrm2728. 19603039

[msaf240-B98] Sun F et al A dual-targeted purple acid phosphatase in *Arabidopsis thaliana* moderates carbon metabolism and its overexpression leads to faster plant growth and higher seed yield. New Phytol. 2012:194:206–219. 10.1111/j.1469-8137.2011.04026.x. 22269069

[msaf240-B99] Sun Q et al PPDB, the plant proteomics database at Cornell. Nucleic Acids Res. 2009:37:D969–D974. 10.1093/nar/gkn654. 18832363 PMC2686560

[msaf240-B100] Terashima M, Specht M, Hippler M. The chloroplast proteome: a survey from the *Chlamydomonas reinhardtii* perspective with a focus on distinctive features. Curr Genet. 2011:57:151–168. 10.1007/s00294-011-0339-1. 21533645

[msaf240-B101] Thagun C, Odahara M, Kodama Y, Numata K. Identification of a highly efficient chloroplast-targeting peptide for plastid engineering. PLoS Biol. 2024:22:e3002785. 10.1371/journal.pbio.3002785. 39298532 PMC11444414

[msaf240-B102] Thoms S . Import of proteins into peroxisomes: piggybacking to a new home away from home. Open Biol. 2015:5:150148. 10.1098/rsob.150148. 26581572 PMC4680570

[msaf240-B103] Thomson SM, Pulido P, Jarvis RP. Protein import into chloroplasts and its regulation by the ubiquitin-proteasome system. Biochem Soc Trans. 2020:48:71–82. 10.1042/BST20190274. 31922184 PMC7054747

[msaf240-B104] Tian L, Okita TW. mRNA-based protein targeting to the endoplasmic reticulum and chloroplasts in plant cells. Curr Opin Plant Biol. 2014:22:77–85. 10.1016/j.pbi.2014.09.007. 25282588

[msaf240-B105] Timmis JN, Ayliff MA, Huang CY, Martin W. Endosymbiotic gene transfer: organelle genomes forge eukaryotic chromosomes. Nat Rev Genet. 2004:5:123–135. 10.1038/nrg1271. 14735123

[msaf240-B106] Tria FDK, Landan G, Dagan T. Phylogenetic rooting using minimal ancestor deviation. Nat Ecol Evol. 2017:1:0193. 10.1038/s41559-017-0193. 29388565

[msaf240-B107] Truscott KN et al A presequence- and voltage-sensitive channel of the mitochondrial preprotein translocase formed by Tim23. Nat Struct Biol. 2001:8:1074–1082. 10.1038/nsb726. 11713477

[msaf240-B108] von Heijne G, Steppuhn J, Herrmann RG. Domain structure of mitochondrial and chloroplast targeting peptides. Eur J Biochem. 1989:180:535–545. 10.1111/j.1432-1033.1989.tb14679.x. 2653818

[msaf240-B109] Von Loeffelholz O et al OEP61 is a chaperone receptor at the plastid outer envelope. Biochem J. 2011:438:143–153. 10.1042/BJ20110448. 21612577 PMC5026492

[msaf240-B110] Waegemann K, Rgen Soll J. 1996. Phosphorylation of the transit sequence of chloroplast precursor proteins. J Biol Chem. 1996;271:6545–6554 10.1074/jbc.271.11.6545. 8626459

[msaf240-B111] Wang C, Youle RJ. The role of mitochondria in apoptosis. Annu Rev Genet. 2009:43:95–118. 10.1146/annurev-genet-102108-134850. 19659442 PMC4762029

[msaf240-B112] Wang Y et al Dual location of the mitochondrial preprotein transporters B14.7 and Tim23-2 in complex I and the TIM17:23 complex in Arabidopsis links mitochondrial activity and biogenesis. Plant Cell. 2012:24:2675–2695. 10.1105/tpc.112.098731. 22730406 PMC3406907

[msaf240-B113] Wang Y, Weiner H. The presequence of rat liver aldehyde dehydrogenase requires the presence of an alpha-helix at its N-terminal region which is stabilized by the helix at its C termini. J Biol Chem. 1993:268:4759–4765. 10.1016/S0021-9258(18)53462-1. 8383124

[msaf240-B114] Weis BL, Schleiff E, Zerges W. Protein targeting to subcellular organelles via mRNA localization. Biochim Biophys Acta Mol Cell Res. 2013:1833:260–273. 10.1016/j.bbamcr.2012.04.004. 23457718

[msaf240-B115] Wunder T, Martin R, Löffelhardt W, Schleiff E, Steiner JM. The invariant phenylalanine of precursor proteins discloses the importance of Omp85 for protein translocation into cyanelles. BMC Evol Biol. 2007:7:236. 10.1186/1471-2148-7-236. 18045484 PMC2222254

[msaf240-B116] Zhang R et al AtPAP2 modulates the import of the small subunit of rubisco into chloroplasts. Plant Signal Behav. 2016:11:e1239687. 10.1080/15592324.2016.1239687. 27700374 PMC5117095

[msaf240-B117] Zhang R et al Overlapping functions of the paralogous proteins atpap2 and atpap9 in *Arabidopsis thaliana*. Int J Mol Sci. 2021:22:7243. 10.3390/ijms22147243. 34298863 PMC8303434

[msaf240-B118] Zhao X, Higa T, Nakai M. Tic12, a 12-kDa essential component of the translocon at the inner envelope membrane of chloroplasts in Arabidopsis. Plant Cell. 2022:34:4569–4582. 10.1093/plcell/koac240. 35929083 PMC9614449

