# Supplementary material for: Evolutionary Refinement of Mitochondrial and Plastid Targeting Sequences Coincides with the Late Diversification of Land Plants
Source: Mol Biol Evol. 2025 Sep 23;42(10):msaf240. doi: 10.1093/molbev/msaf240 (PMC12531127; doi:10.1093/molbev/msaf240)
Supplement: msaf240_Supplementary_Data [file msaf240_supplementary_data.pdf]

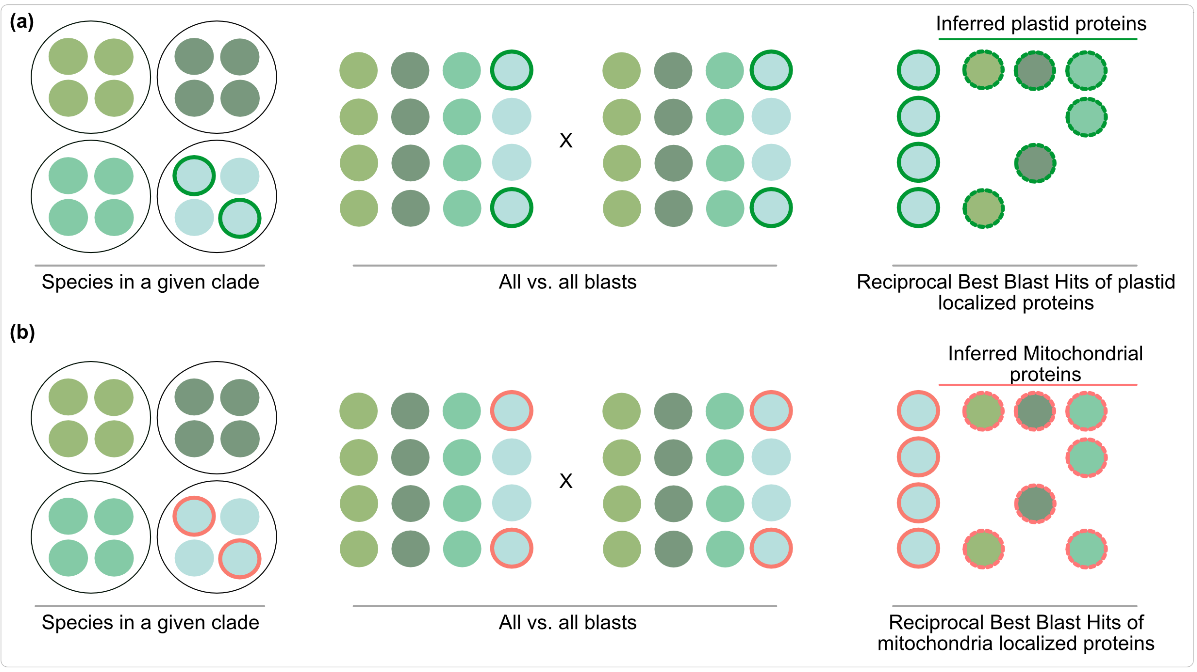

Fig. S1: Inferring organelle localised proteins across five major chloroplastida clades. 110 Archaeplastida species of chlorophyte and streptophyte algae, bryophytes, monocots and eudicot clades were used for the analysis. Each black circle outline represents a species and the smaller, coloured dots represent individual proteins. Dots with coloured outlines represent experimentally validated plastid (green outline) and mitochondrial (salmon outline) proteins. All vs. all blasts were conducted for all proteins, from all species within each clade. Reciprocal best Blast hits (RBBH) were obtained for all experimentally validated **(a)** plastid- and **(b)** mitochondria-localised proteins in a given species to infer organelle localised proteins (dotted outlines, on the right) for all species in that clade.

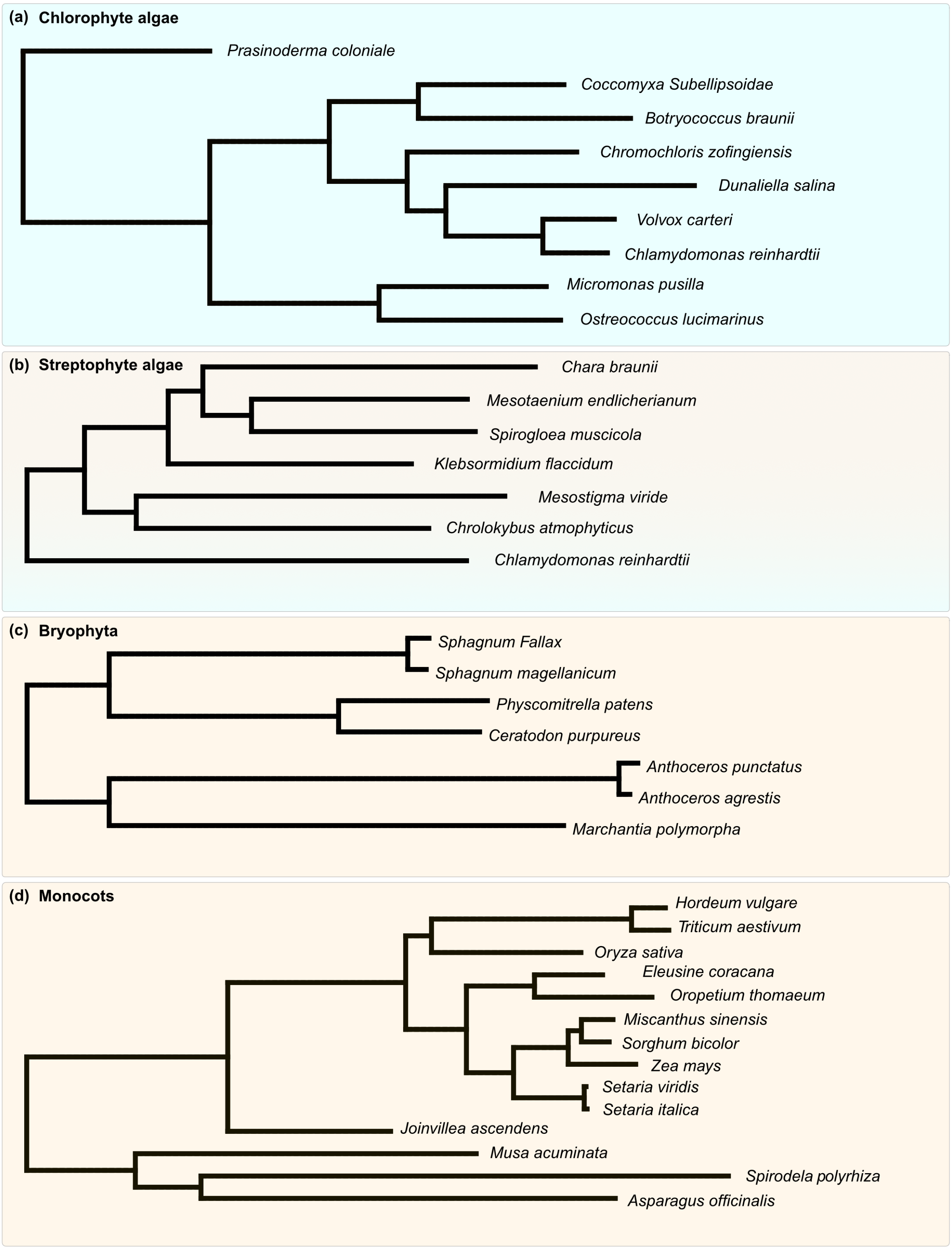

(a) Chlorophyte algae
Prasinoderma coloniale
Coccomyxa Subellipsoidae
Botryococcus braunii
Chromochloris zofingiensis
Dunaliella salina
Volvox carteri
Chlamydomonas reinhardtii
Micromonas pusilla
Ostreococcus lucimarinus
(b) Streptophyte algae
Chara braunii
Mesotaenium endlicherianum
Spirogloea muscicola
Klebsormidium flaccidum
Mesostigma viride
Chrolokybus atmophyticus
Chlamydomonas reinhardtii
(c) Bryophyta
Sphagnum Fallax
Sphagnum magellanicum
Physcomitrella patens
Ceratodon purpureus
Anthoceros punctatus
Anthoceros agrestis
Marchantia polymorpha
(d) Monocots
Hordeum vulgare
Triticum aestivum
Oryza sativa
Eleusine coracana
Oropetium thomaeum
Miscanthus sinensis
Sorghum bicolor
Zea mays
Setaria viridis
Setaria italica
Joinvillea ascendens
Musa acuminata
Spirodela polyrhiza
Asparagus officinalis

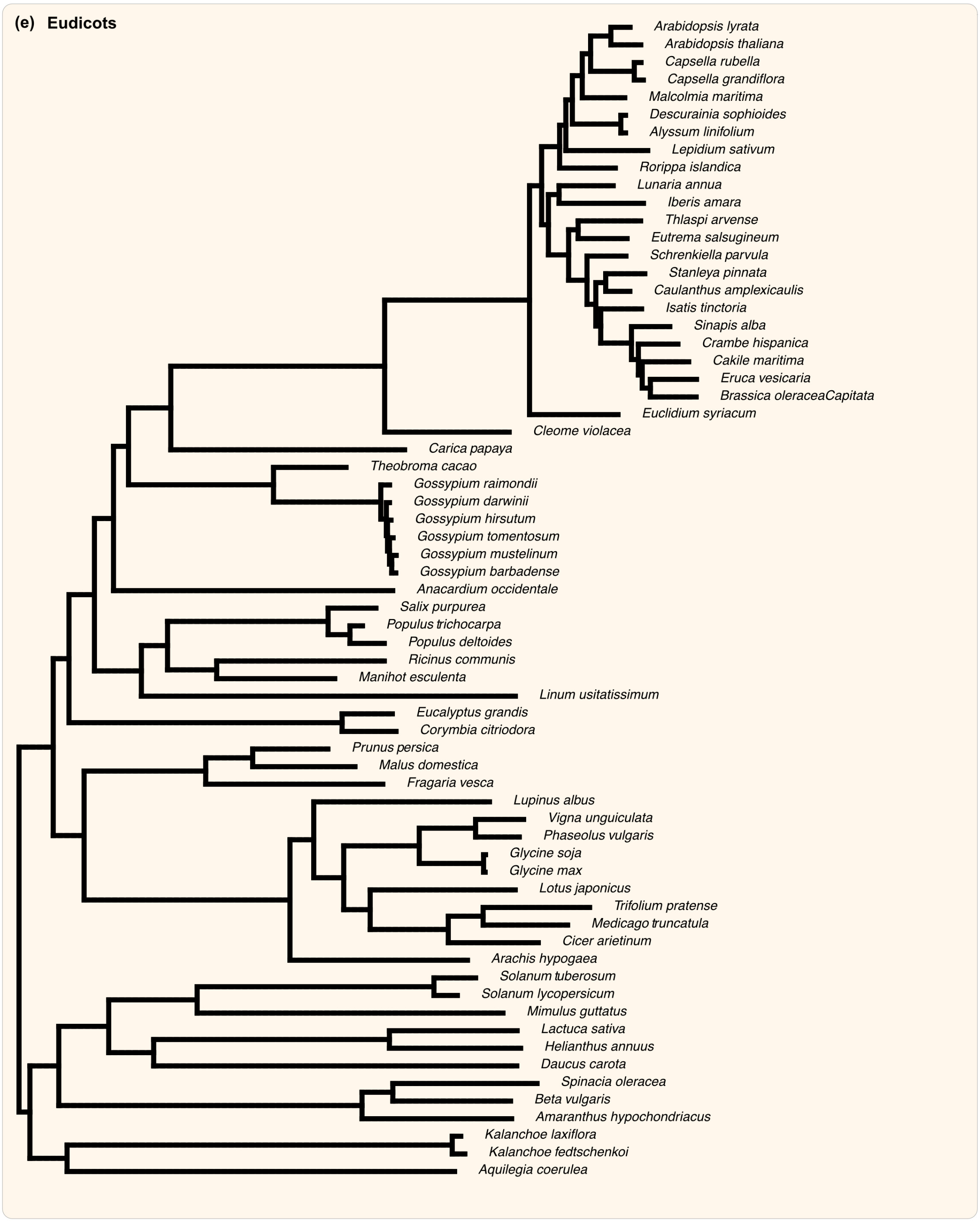

Fig. S2: Phylogenies of the five major chloroplastidal clades. Inferred phylogenies of each clade based on the concatenation of organelle localised proteins present in all species for that clade.

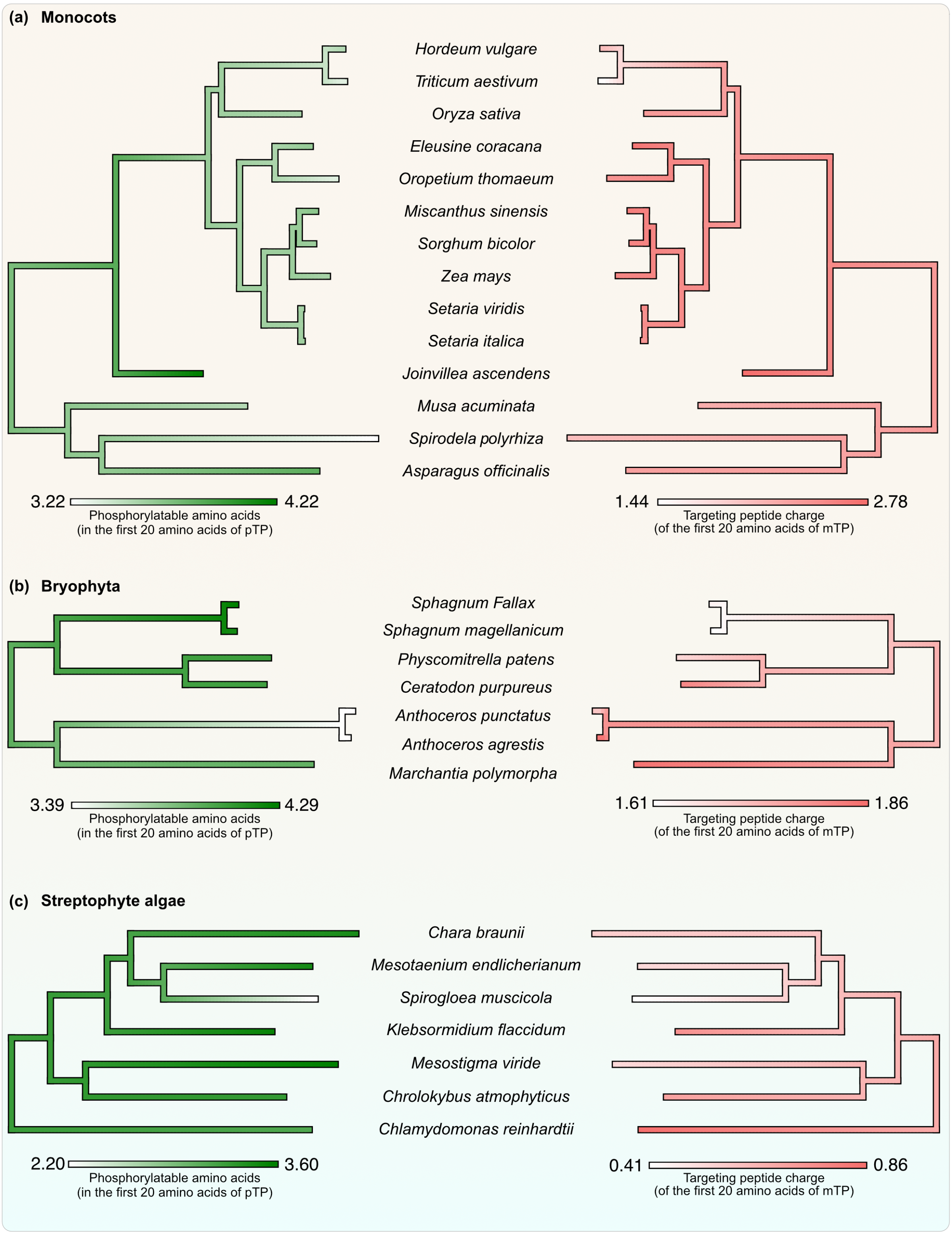

Fig. S3: Ancestral states of phosphorylatable amino acids in ps and charges of ms, inferred across ancestors of **(a)** monocots, **(b)** bryophytes and **(c)** streptophyte algae, color-coded based on the values of the two features across ancestors.

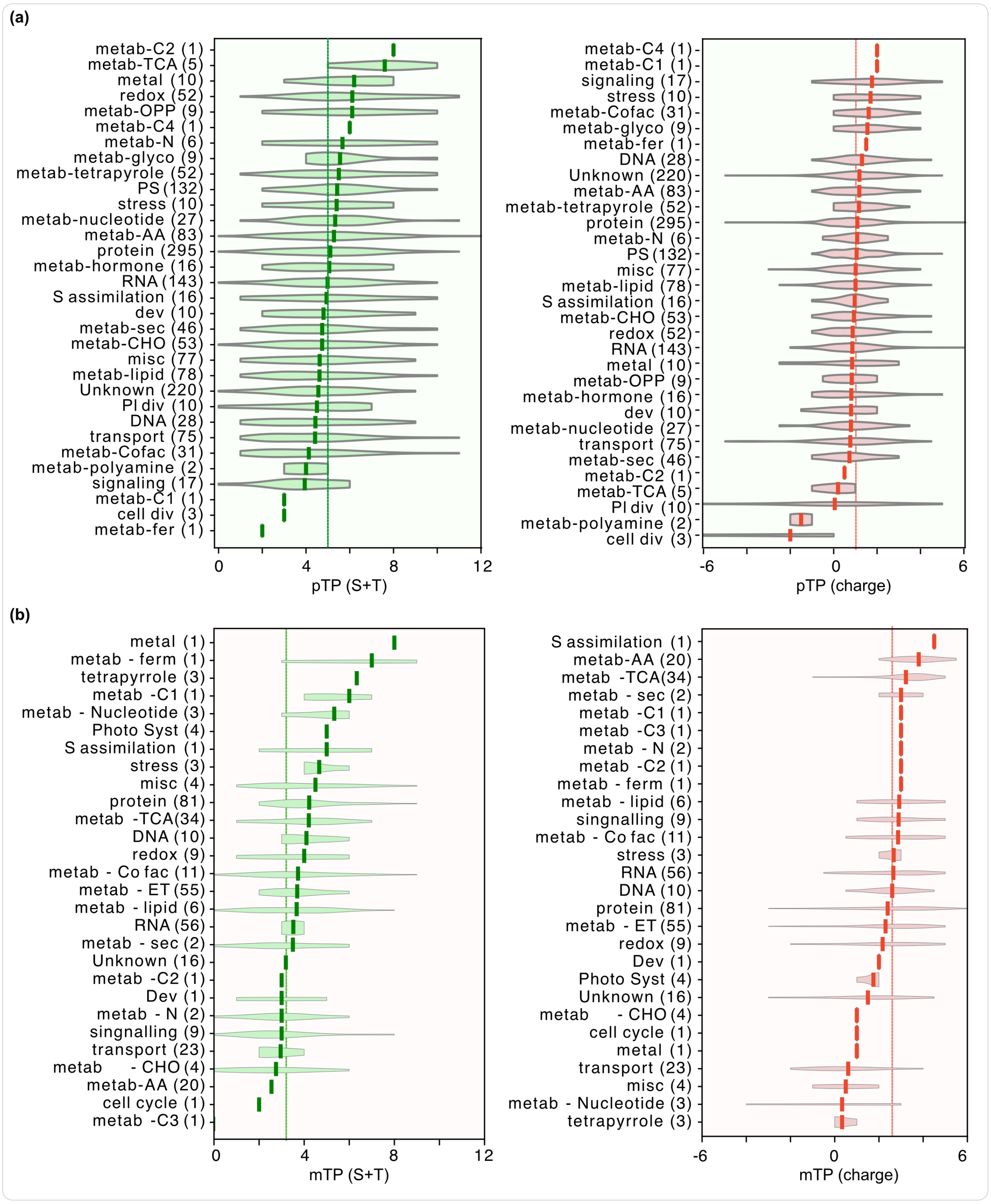

Fig. S4: The distribution of the number of phosphorylatable amino acids and charge in the first 20 amino acids of **(a)** plastid and **(b)** mitochondrial proteins in *Arabidopsis*, in each functional category (shown on the left, with number of total proteins in the parenthesis). The vertical lines (running across functional categories) indicate average values for all proteins in that organelle. For full names of the functional categories, please refer to the figure source data.

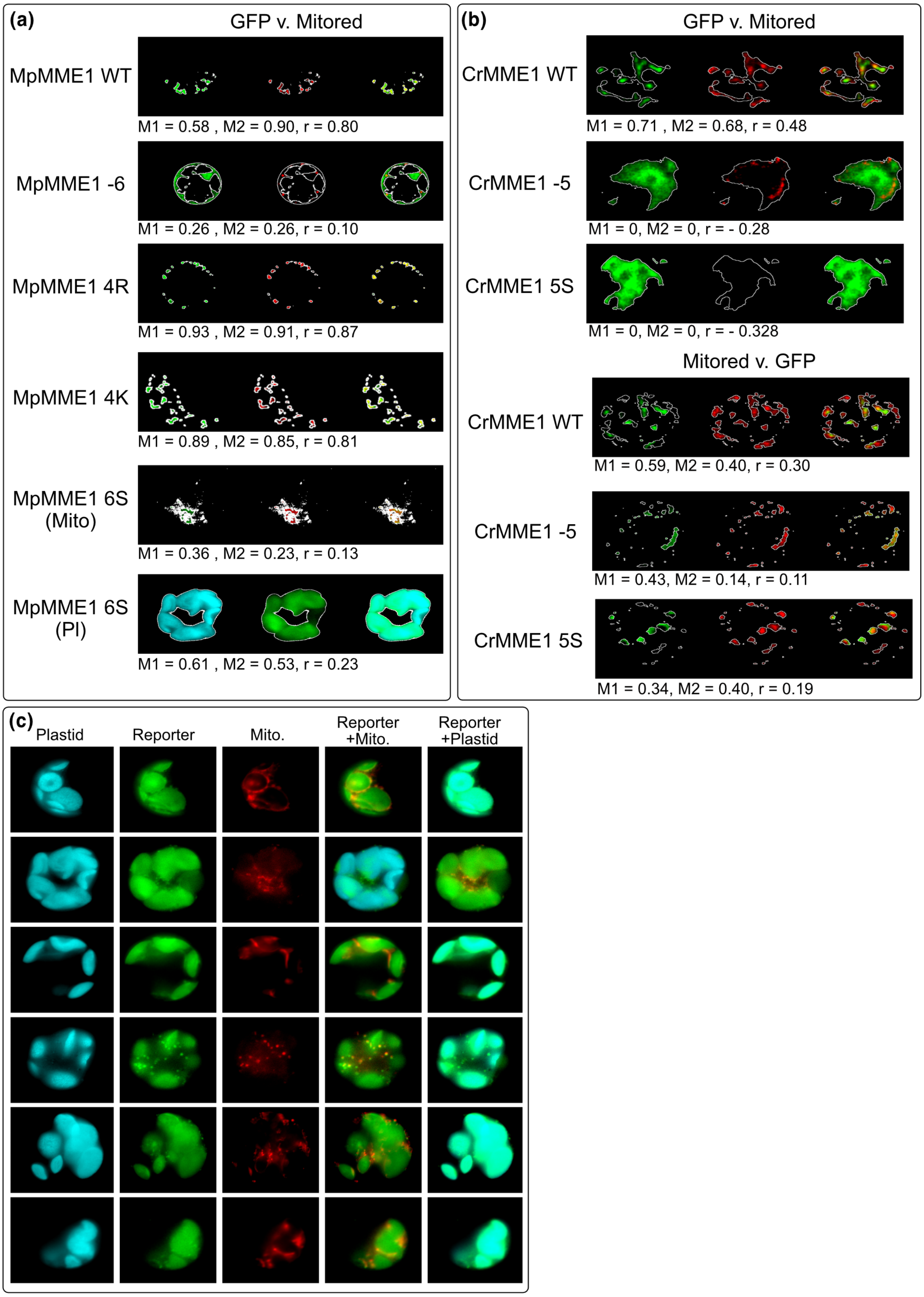

Fig. S5: Additional evidence for the colocalisation and dual targeting of constructs in *Marchantia* and *Chlamydomonas*. **(a)** Colocalisation analyses for *Marchantia* MME1 constructs shows the mitochondrial signal localised within the GFP signal. Mander's overlap coefficients M1 and M2, respectively, show a fraction of the reporter signal overlapping with mitochondria and *vice versa* (e.g. M1= 0.58 and M2=0.90, which means 58% of the GFP region is stained with mitochondria and in 90%

of the mitochondrial region GFP is observed (due to the membrane potential dependent nature of the dye, mitochondria are not completely stained). R shows the correlation coefficient for intensities of the two channels. **(b)** Colocalization analyses for *Chlamydomonas* MME1 constructs with the total GFP region and the total mitochondrial region as region of interests. **(c)** Different protoplasts from the same MME1 6S constructs, all further validating the dual targeting.

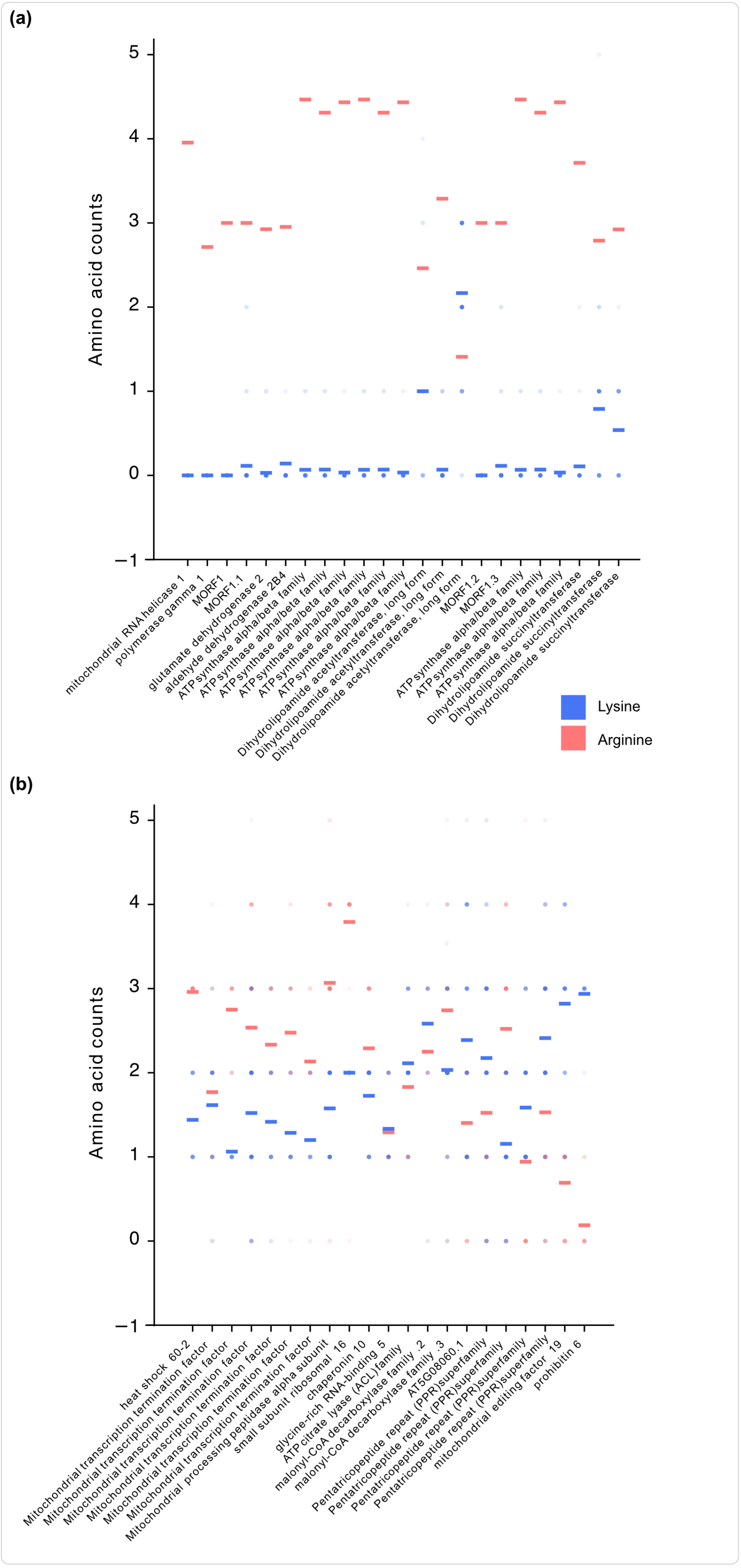

Fig. S6: Arginine (R) vs. lysine (K) preference across mTPs of 72 eudicots. R and K counts from the first 20 amino acids for orthologues of *Arabidopsis* mitochondrial proteins with a charge >3. Proteins showing the strongest preference for **(a)** R and **(b)** K are plotted. Each column is a protein; each data point is a number of R (salmon) or K (blue) for a given species. The horizontal lines indicate the average R (in salmon) and K numbers (in blue) for that protein's orthologues across eudicots.

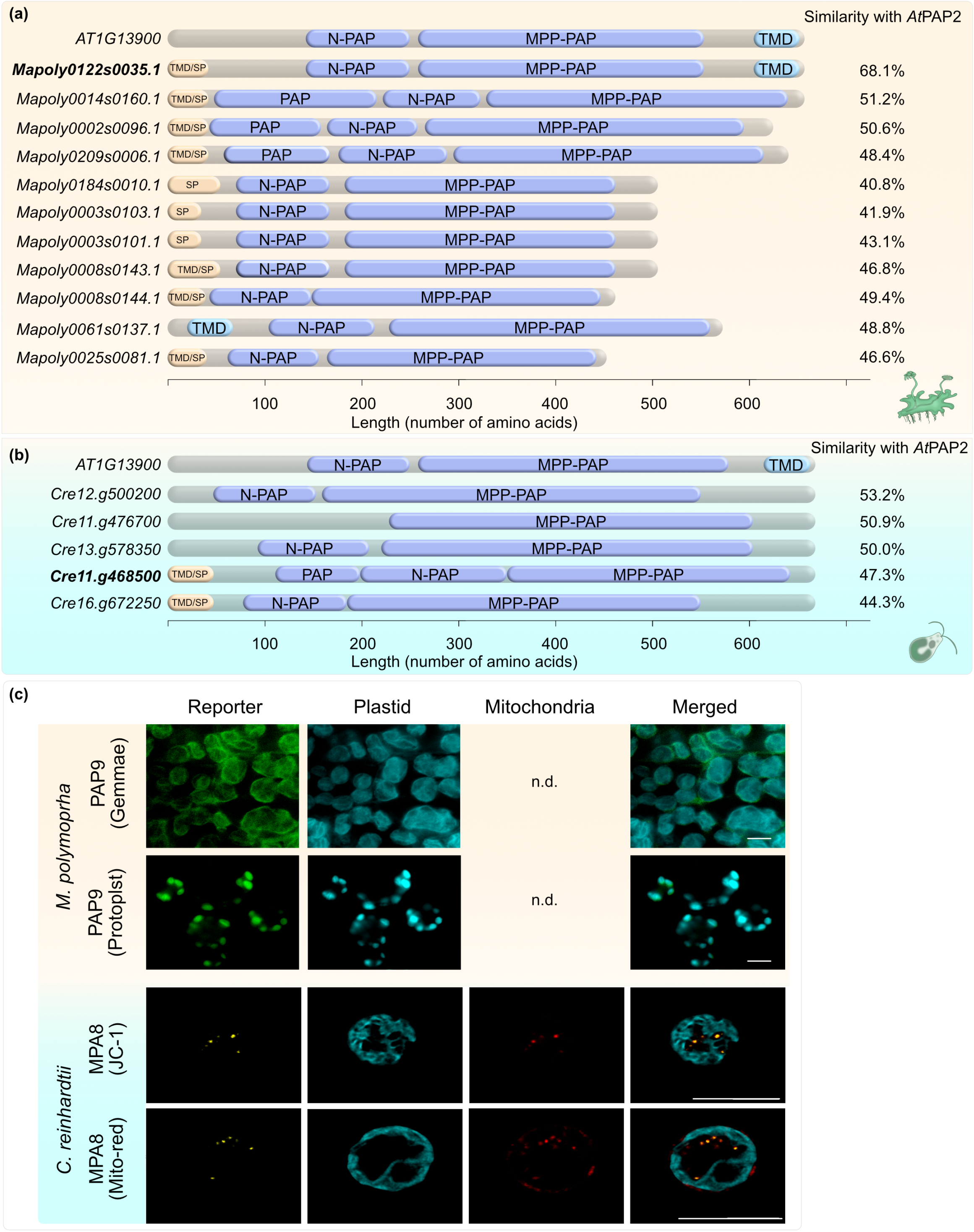

Fig. S7: Identification and localisation of PAP2/9 homologues in *Chlamydomonas* and *Marchantia*. *Arabidopsis* PAP2 (AT1G13900) has five homologues in *Chlamydomonas reinhardtii* (genome v5.6). **(a)** Their functional and membrane docking domain analyses narrowed down Cre11.g468500 as the best match, as it has the signature PAP2 domains, as well as a membrane anchoring/signalling peptide with a probability higher than 0.5. The functional domains (N-terminal purple acid phosphatase, N-PAP; metalloprotein phosphatase, MPP-PAP) were inferred via InterProScan (https://www.ebi.ac.uk/interpro/). Transmembrane domains (TMD) were initially inferred via TMHMM2 (https://services.healthtech.dtu.dk/services/TMHMM-2.0/). The updated version deepTMHMM (https://dtu.biolib.com/DeepTMHMM) annotated the same region in some cases (e.g. the N-terminal region in *Chlamydomonas*) as a signal peptide (SP), and we therefore labelled those

regions ‘TMD/SP’. Regions with a single label ‘TMD’ (e.g. at the C-terminus of *At*PAP2) are the ones where TMHMM2 and deepTMHMM both annotated it as TMD. **(b)** Similar to *C. reinhardtii,* all *M. polymoprha* v3.1 homologues were analysed to annotate Mapoly0122s0035 as the best candidate for *Mp*PAP. **(c)** N-terminus of *Cr*MPA8 and the full length *Mp*PAP9 were localized in the thallus and protoplasts of *Marchantia*, and in cells of *Chlamydomonas.* Plastids were imaged through their autofluorescence and mitochondria through MitoTracker Red and JC-1. Scalebars 5µM.

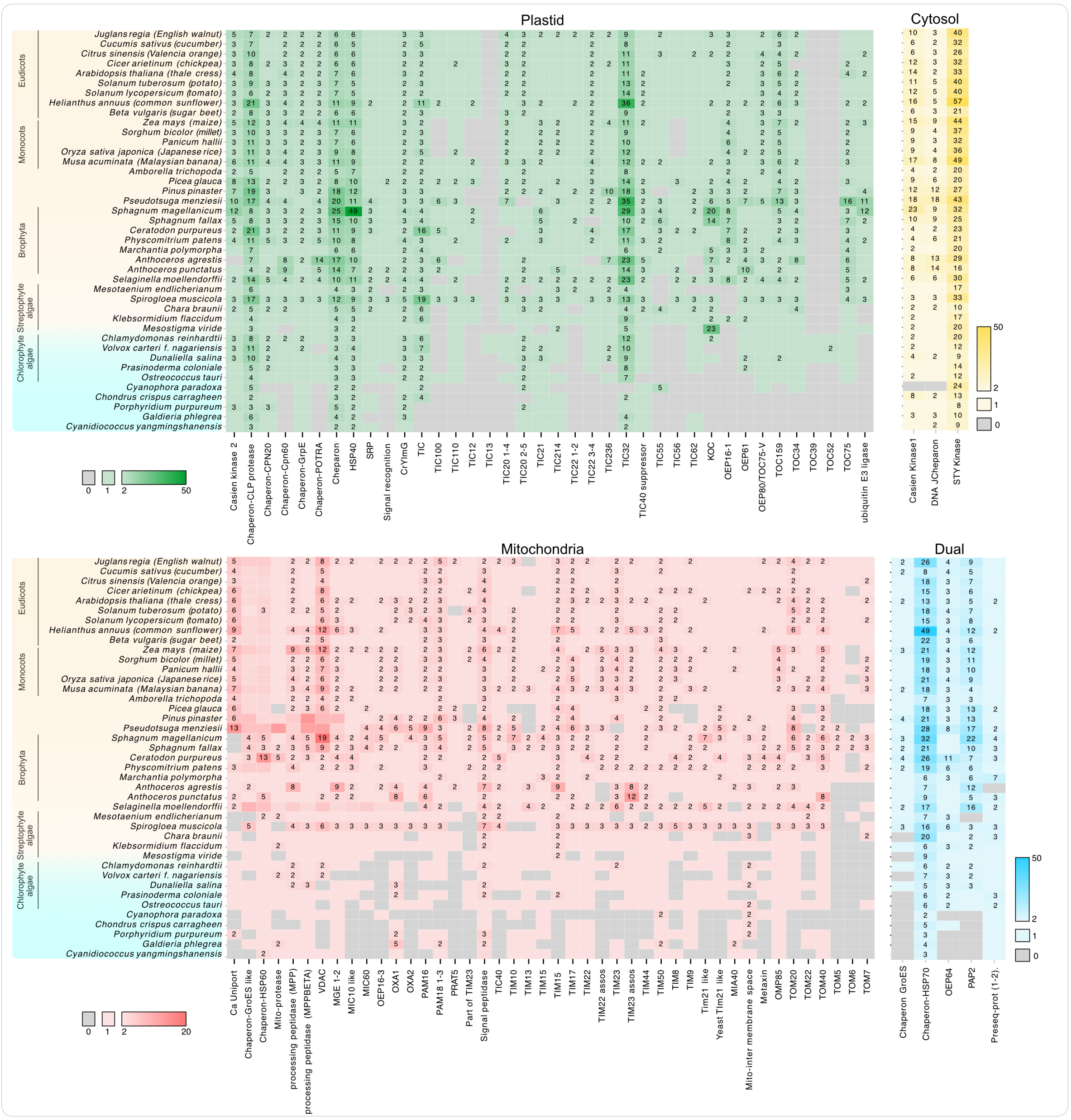

Fig. S8: Gene copy numbers (GCN) of import components across the Archaeplastida. Entries are separated based on their single intracellular localisation (plastid, cytosol, or mitochondrial) or dual (plastid and mitochondrial). Colour schemes represent the GCN; components with more than one copy are shown also by the values on each cell.

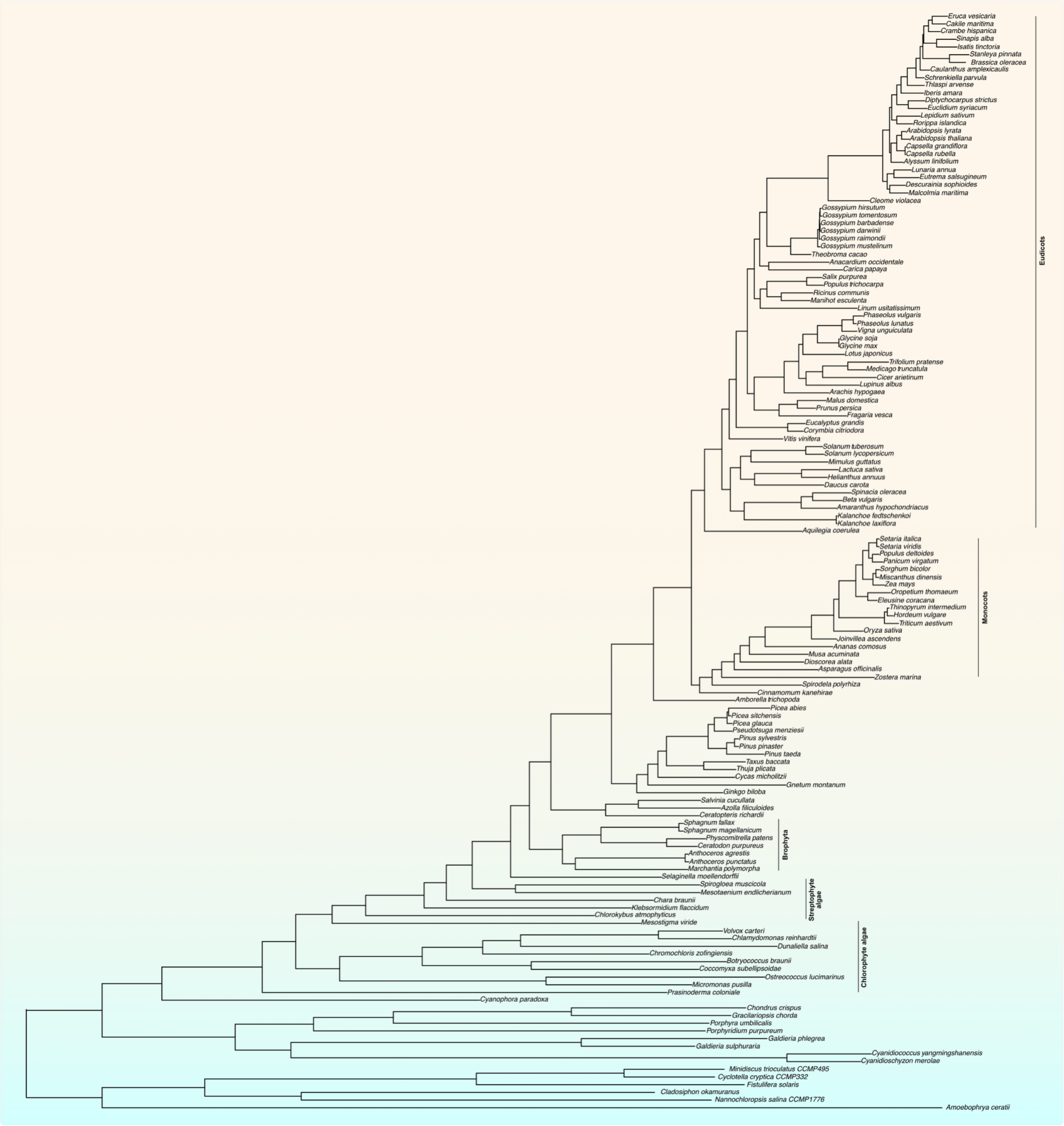

Fig. S9: Phylogeny of 137 archaeplastidal species (with six species as an outgroup).

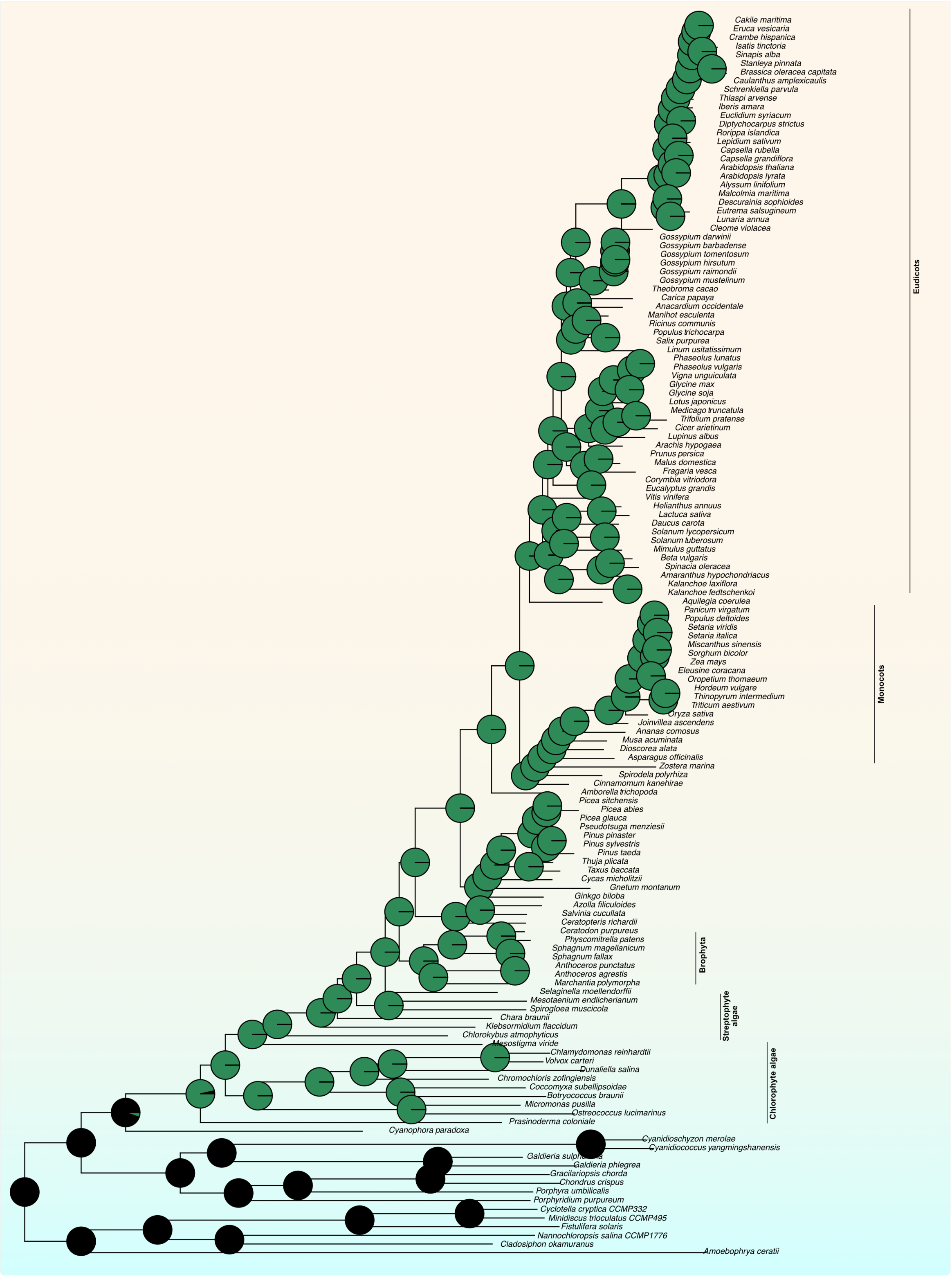

Fig. S10: Ancestral state reconstruction for TIC56, i.e. the full phylogeny and ASR of the detail in Fig. 5B.

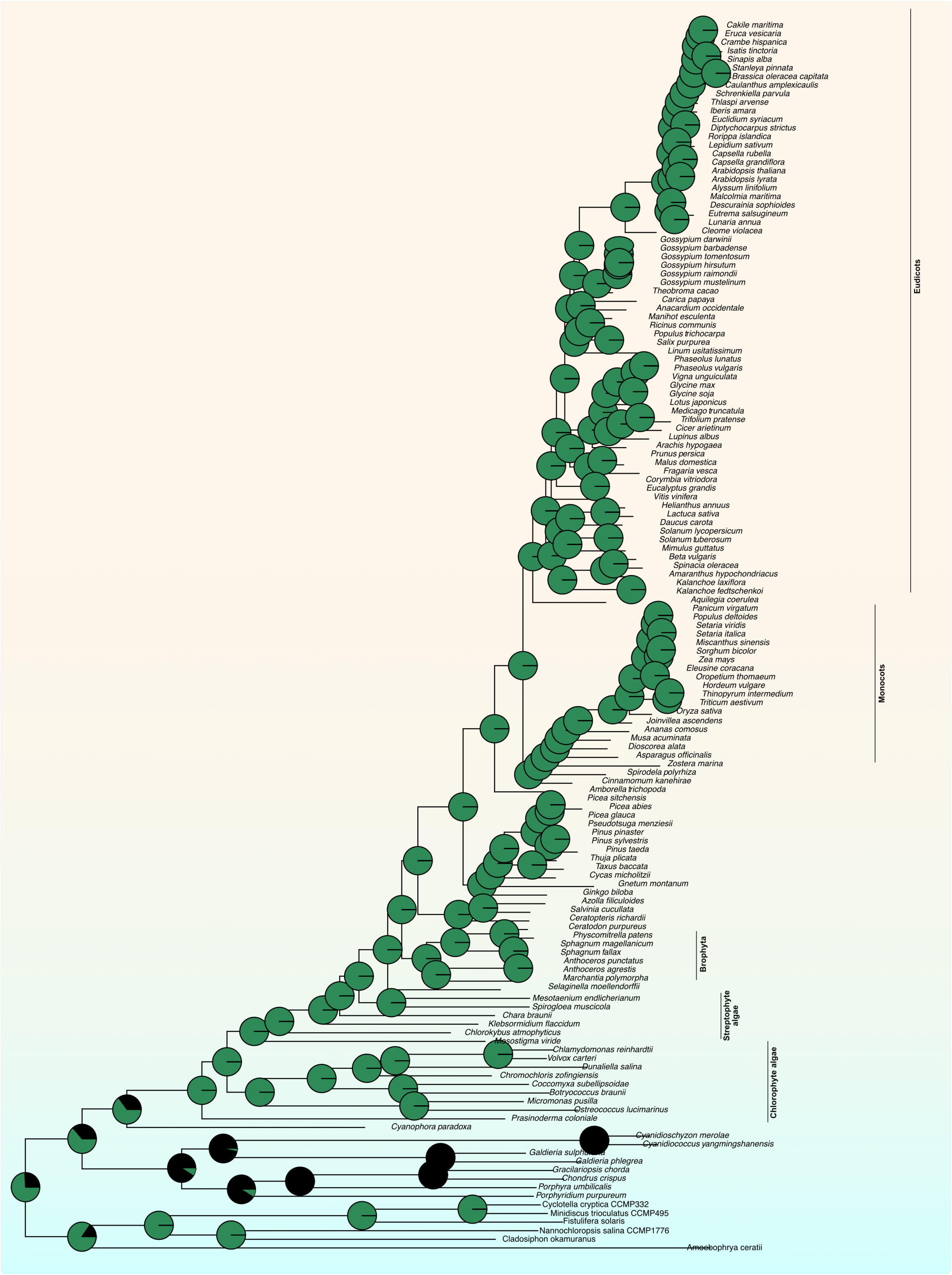

Fig. S11: Ancestral state reconstruction for TIC100, i.e. the full phylogeny and ASR of the detail in Fig. 5B.

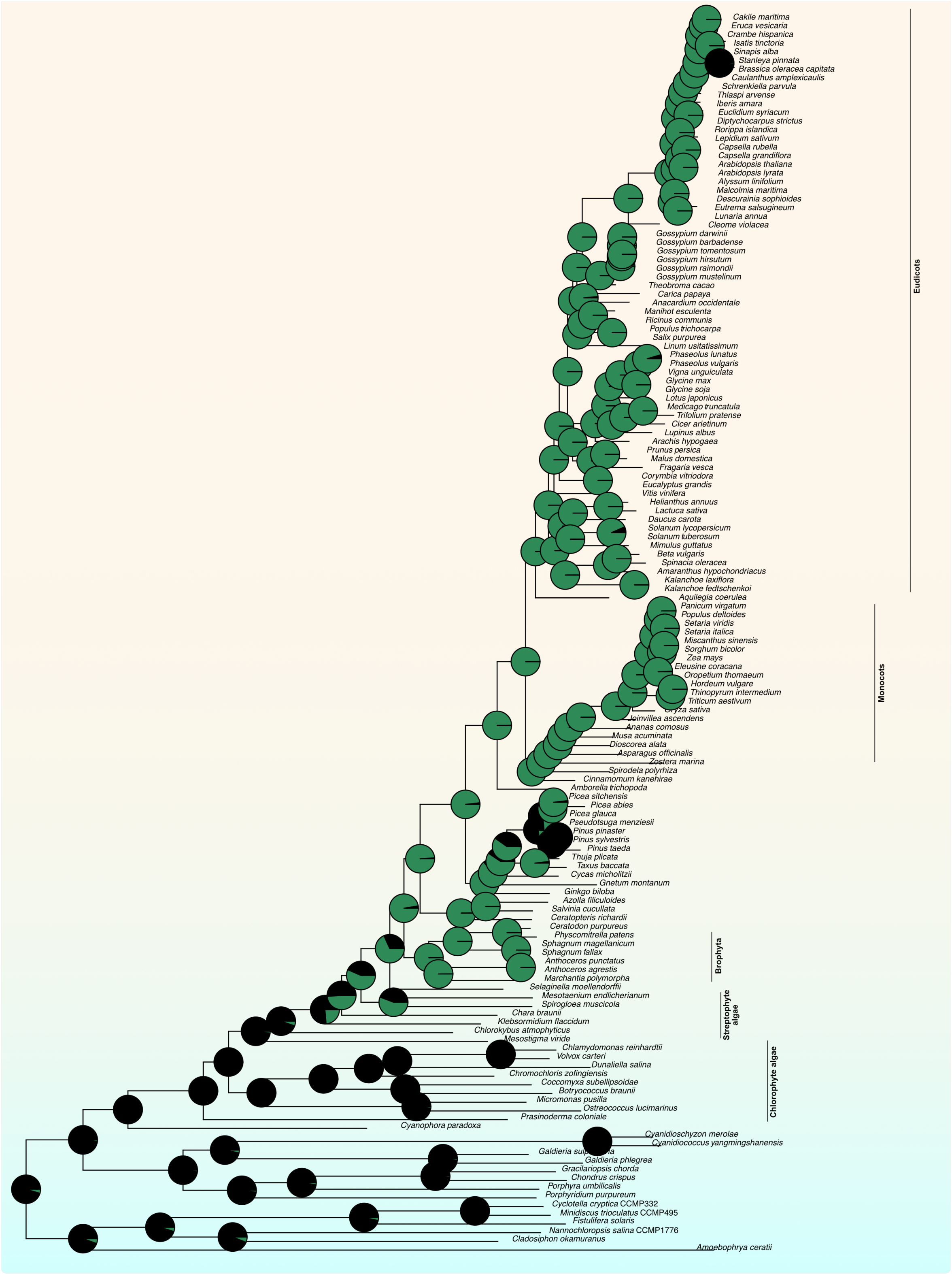

Fig. S12: Ancestral state reconstruction for TIC12, i.e. the full phylogeny and ASR of the detail in Fig. 5B.

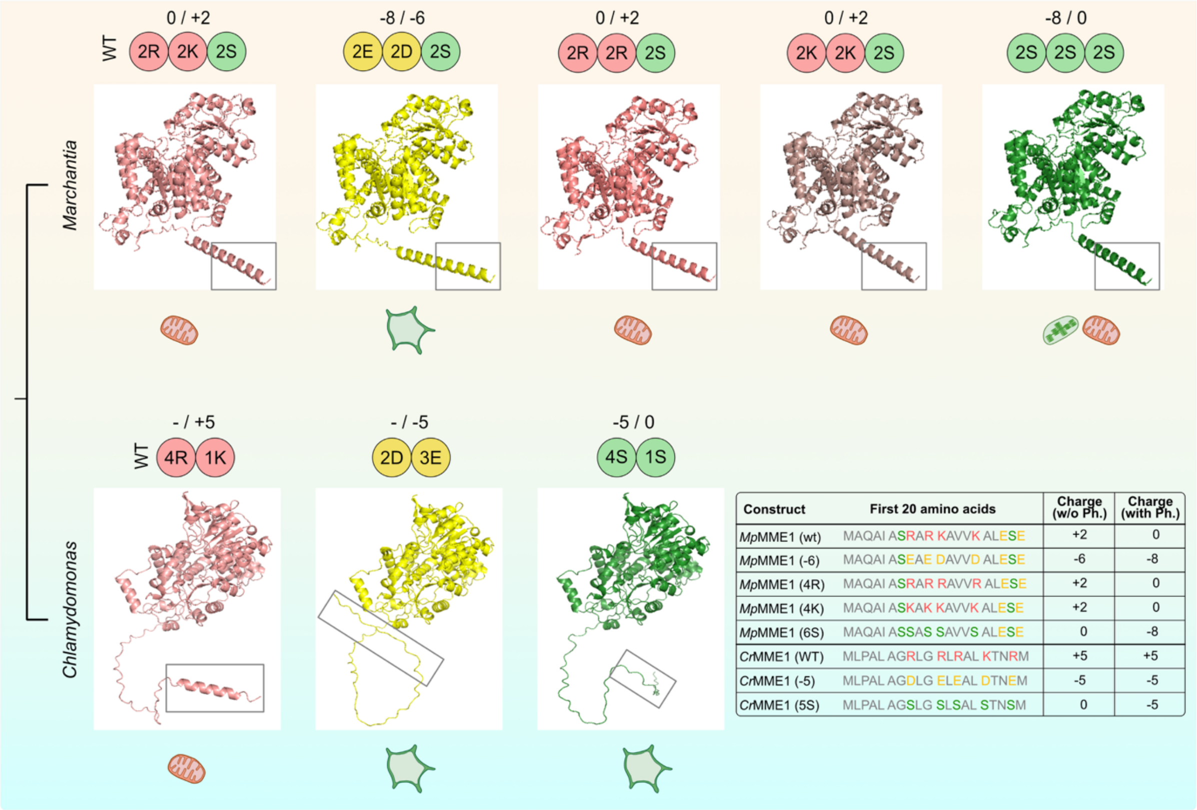

Construct	First 20 amino acids	Charge (w/o Ph.)	Charge (with Ph.)
*Mp*MME1 (wt)	MAQAI ASRAR KAVVK ALESE	+2	0
*Mp*MME1 (-6)	MAQAI ASEAE DAVVD ALESE	-6	-8
*Mp*MME1 (4R)	MAQAI ASRAR RAVVR ALESE	+2	0
*Mp*MME1 (4K)	MAQAI ASKAK KAVVK ALESE	+2	0
*Mp*MME1 (6S)	MAQAI ASSAS SAVVS ALESE	0	-8
*Cr*MME1 (WT)	MLPAL AGRLG RLRAL KTNRM	+5	+5
*Cr*MME1 (-5)	MLPAL AGDLG ELEAL DTNEM	-5	-5
*Cr*MME1 (5S)	MLPAL AGSLG SLSAL STNSM	0	-5

Fig. S13: Structural predictions for MME1 constructs used in the study and their observed localisation (depicted by icons for mitochondria, plastids or cytosol). The key amino acids (in single letter codes) are shown on top of each structure along with the resulting charge of the first 20 amino acids (with/without phosphorylation). See the table on the bottom right for the full sequence of the first 20 amino acids and resulting charges. The first 20 amino acids are highlighted by a box in each structure prediction.

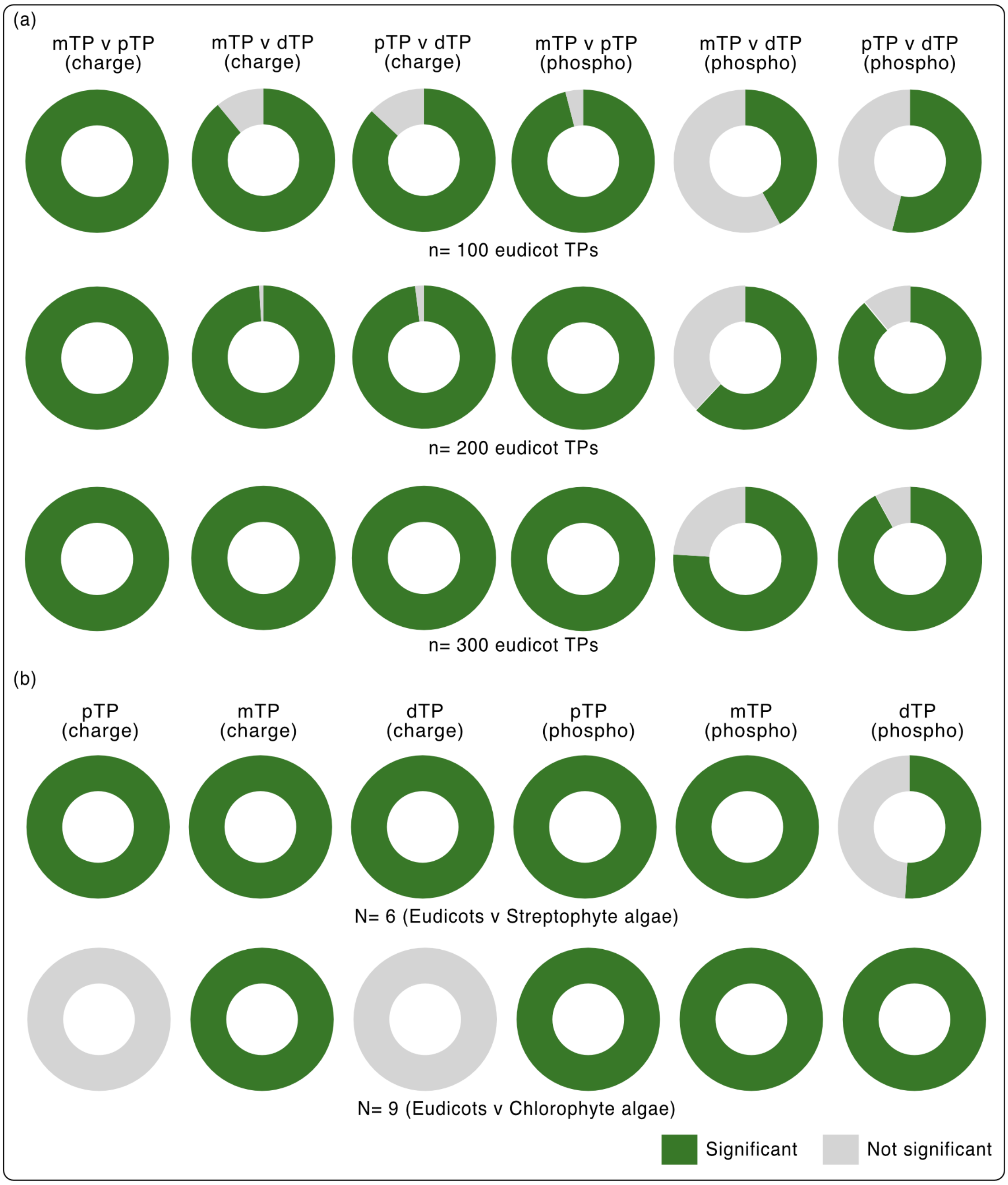

Fig. S14: Subsampling of the number of TPs (n) and number of species (N) at the lowest end of sample sizes. The sample sizes for N and n are both skewed towards specific organelles and clades, which could affect inferences across clades and on the organelle divergence of TPs. **(a)** To test whether charge and phosphorylation of eudicot mTP and pTP are significantly different under a low TP count (n), we subsampled 100, 200 and 300 TPs for each organelle and undertook an all-v-all comparisons. Instances where the two groups of TPs were significantly different (Mann Whitney U test), out of 100 such subsampling, are plotted as donut plots. **(b)** To test whether the charge and phosphorylatable amino acids are significantly different between algae and eudicots for a given TP class (e.g. mTP), we subsampled 6 eudicot species and compared them against the 6 available streptophyte algae and likewise, compared 9 eudicots against 9 available chlorophyte algae. Statistically significant instances (Mann Whitney U test) out of 100 such subsamples are plotted.

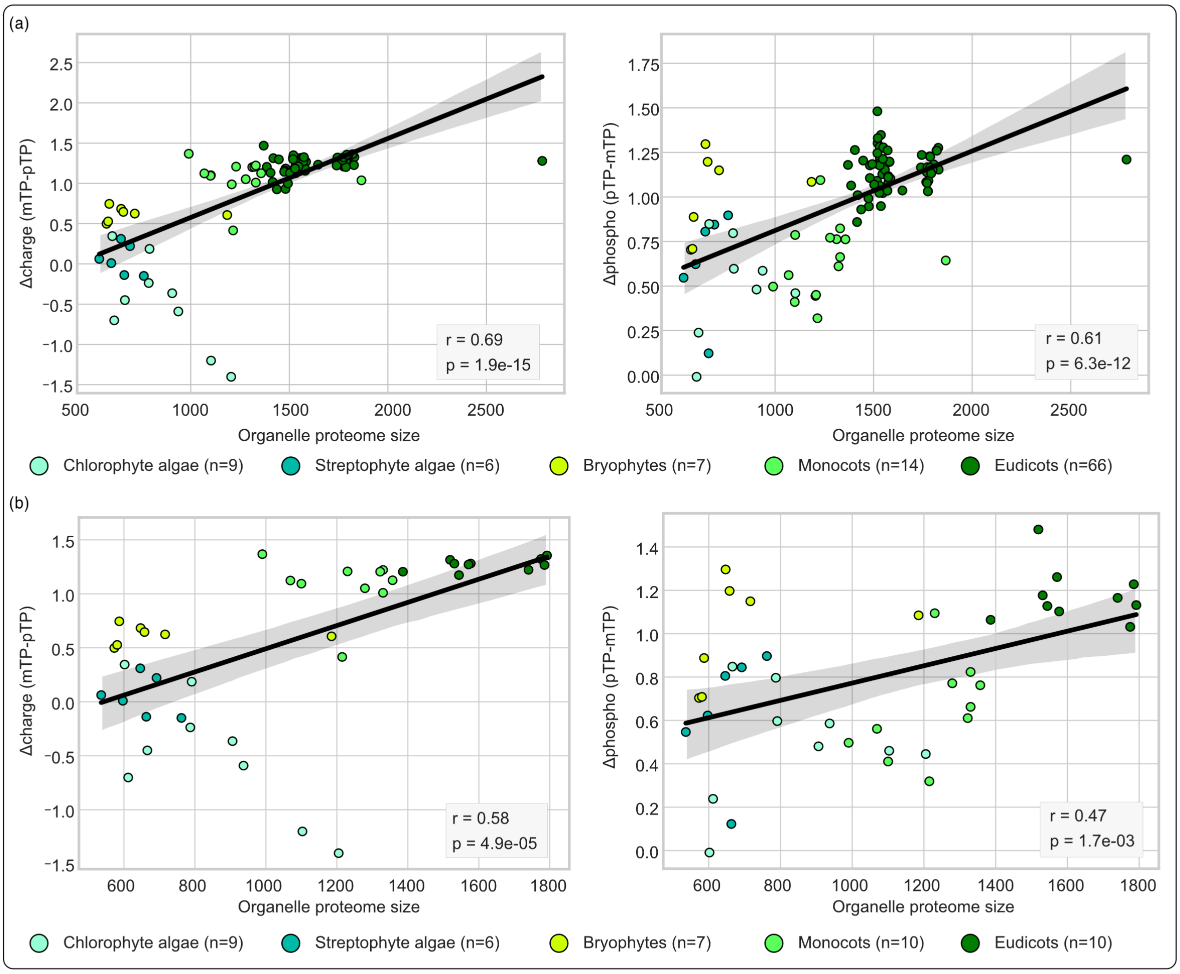

Fig. S15: Organelle proteome size and TP divergence. To test for a correlation between organelle proteome size (number of plastidal + mitochondrial proteins for each species) and TP divergence, the TP divergence was proxied by Δcharge (mTP-pTP) and Δphospho (pTP-mTP). Δcharge was calculated for each species (each circle on the left-side plots) as a difference between mean charge of all mTPs of that species and mean charge of all pTPs of the same species. Δphospho was calculated for each species (each circle on the right-side plots) as a difference between mean phospho of all pTPs of that species and mean phospho of mTPs of the same species. **(a)** Correlation across all species from all clades (n, number of species per clade is shown at the bottom of the figure). **(b)** To normalize for higher samples size of monocots and eudicots, the same correlations were repeated with 10 randomly subsampled species from monocot and eudicot clades and all species from chlorophyte algae, streptophyte algae, bryophyte (n, number of species per clade is shown at the bottom of the figure).
